# ST6GalNAc-I regulates tumor cell sialylation via NECTIN2/MUC5AC-mediated immunosuppression and angiogenesis in non–small cell lung cancer

**DOI:** 10.1172/JCI186863

**Published:** 2025-05-15

**Authors:** Muthamil Iniyan Appadurai, Sanjib Chaudhary, Ashu Shah, Gopalakrishnan Natarajan, Zahraa W. Alsafwani, Parvez Khan, Dhananjay D. Shinde, Subodh M. Lele, Lynette M. Smith, Mohd Wasim Nasser, Surinder Kumar Batra, Apar Kishor Ganti, Imayavaramban Lakshmanan

**Affiliations:** 1 Department of Biochemistry and Molecular Biology;; 2 Department of Pathology, Microbiology, and Immunology;; 3 Department of Pathology and Microbiology;; 4 Department of Biostatistics, College of Public Health;; 5 Fred & Pamela Buffett Cancer Center; and; 6 Department of Internal Medicine, University of Nebraska Medical Center, Omaha, Nebraska, USA.; 7 Division of Oncology-Hematology, Department of Internal Medicine, VA Nebraska-Western Iowa Health Care System, Omaha, Nebraska, USA.

**Keywords:** Cell biology, Immunology, Oncology, Angiogenesis, Glycobiology, Lung cancer

## Abstract

Glycosylation controls immune evasion, tumor progression, and metastasis. However, how tumor cell sialylation regulates immune evasion remains poorly characterized. ST6GalNAc-I, a sialyltransferase that conjugates sialic acid to the glycans in glycoproteins, was overexpressed in an aggressive-type KPA (*Kras^G12D/+^ Trp53^R172H/+^ Ad-Cre*) lung adenocarcinoma (LUAD) model and patient samples. Proteomic and biochemical analysis indicated that ST6GalNAc-I mediated NECTIN2 sialylation in LUAD cells. ST6GalNAc-I–deficient tumor cells cocultured with T cells were more susceptible to T cell–mediated tumor cell killing, indicating a key role for NECTIN2 in T cell dysfunction. Mice injected with St6galnac-I–knockdown syngeneic cells showed reduced lung tumor incidence and Nectin2/Tigit-associated immunosuppression. ST6GalNAc-I–deficient cells exhibited reduced P-DMEA metabolite levels, while administration of P-DMEA promoted LUAD cell proliferation via MUC5AC. MUC5AC interacted and colocalized with PRRC1 in the Golgi, suggesting a potential role for PRRC1 in MUC5AC glycosylation. Mice injected with ST6GalNAc-I/MUC5AC-deficient cells (human LUAD) exhibited reduced lung tumor incidence, angiogenesis, and liver metastases. Mechanistically, ST6GalNAc-I/MUC5AC regulates VCAN-V1, a key factor in tumor matrix remodeling during angiogenesis and metastasis. These findings demonstrate that ST6GalNAc-I–mediated sialylation of NECTIN2/MUC5AC is critical for immune evasion and tumor angiogenesis. Targeting this pathway may prevent LUAD development and/or metastasis.

## Abstract 2

ST6GalNAc-I promotes immune evasion and angiogenesis through NECTIN2/MUC5AC sialylation in lung adenocarcinoma development

## Graphical abstract

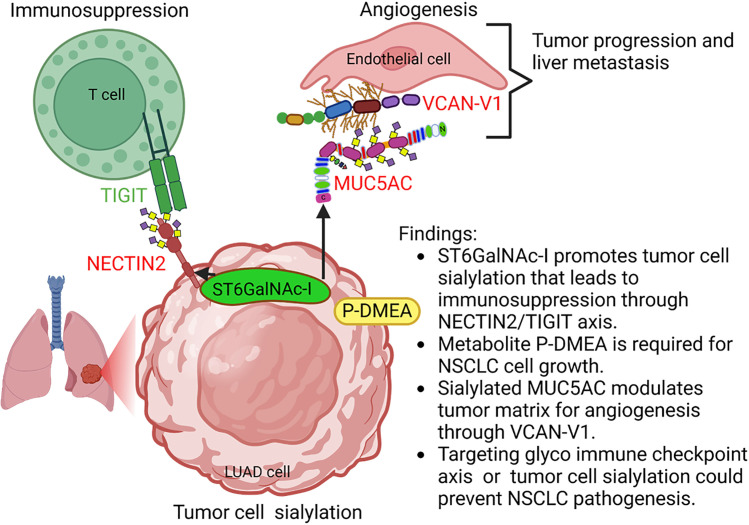

## Introduction

Lung cancer is the most common cause of cancer-related deaths worldwide (1). In the United States, there will be an estimated 226,650 new lung cancer cases that will constitute approximately 21% of all cancer-related deaths in 2025 (2). Despite recent advances, the overall 5-year survival remains poor because of the malignancy’s being diagnosed at an advanced stage (2, 3).

Tumor cell glycosylation controls many cell signaling functions, and the altered glycosylation pattern contributes to disease progression, metastasis, and evasion of the immune responses (4, 5). Several studies have demonstrated that aberrant sialylation (sialyl-Tn [STn]) modulates the immunosuppressive and vascular landscape during tumor progression and metastasis, which contributes to poor survival of patients with lung adenocarcinoma (LUAD) (6–8). Sialylation is the terminal addition of sialic acid to glycans, including glycoproteins, proteoglycans, and glycolipids (9, 10). ST6GalNAc-I (*N*-acetylgalactosaminide α2,6-sialyltransferase 1) is an *O*-glycosyltransferase, which conjugates sialic acid with an α2,6 linkage to *N*-acetylgalactosamine (GalNAc) glycans, generating the tumor-related STn antigen (6, 11, 12). Understanding immune suppression mediated by tumor cell sialylation and its impact on the vascular landscape of non–small cell lung cancer is essential to identifying alternative therapeutic strategies for LUAD.

We and others have shown that ST6GalNAc-I is overexpressed both in human (13–15) and in genetically engineered mouse LUAD tumors (*Kras^G12D/+^ Trp53^R172H/+^* *Ad-Cre* [KPA]) (15). We previously discovered that ST6GalNAc-I–induced sialylation of the mucin MUC5AC is required for LUAD cell growth and metastasis (15). Typically, mucins undergo glycosylation predominantly in their central domain, rich in serine and threonine residues (sites for *O*-glycosylation) (16, 17). Aberrantly glycosylated mucins are critical in tumor progression and metastasis (18, 19). We have identified that the secretory mucin MUC5AC interacts with β_4_ integrin to mediate migration/invasion through FAK (Y397) (20). We have also demonstrated that MUC5AC is overexpressed in LUAD and is associated with a poor prognosis (20). In addition, MUC5AC-deficient lung tumors show reduced tumor angiogenesis, suggesting that MUC5AC is involved in angiogenesis (21). Tumors are prone to immune escape by developing strategies to evade immunity (22). Proline-rich coiled-coil 1 (PRRC1) is preferentially localized in the endoplasmic reticulum–Golgi exit and modulates membrane association of COPII coat, where it regulates ER-to-Golgi transport of secretory and membrane proteins (23).

Nectin cell adhesion molecule 2 (NECTIN2) is an immunoglobulin-like glycoprotein that plays a role in trans-interactions and modulation of cell-cell contact during tumor progression (24). Recent studies have demonstrated that cancer cells expressing NECTIN2 inhibit T and natural killer (NK) cell function by interacting with T cell immunoglobulin and ITIM domain (TIGIT) (25, 26). TIGIT is an inhibitory receptor expressed on the surface of CD4^+^ and CD8^+^ T cells and NK cells, and it is targeted in cancer (27).

Versican (VCAN) is a large chondroitin sulfate/dermatan sulfate proteoglycan predominantly expressed during early lung development but is seen in very low levels in normal adult lungs (28–30). Alternative splicing of mRNA encoding the glycosaminoglycan domain of the VCAN core generates 4 common splice isoforms (V0, V1, V2, and V3) (31, 32). VCAN-homozygous mutant embryos lacking all VCAN isoforms die by 10.5 days of gestation because of immature vasculature (33), suggesting the requirements of VCAN and its isoforms for vascular development.VCAN-V1 and its proteolytic fragment versikine are involved in the angiogenesis and metastasis of various cancers (31, 34–36). VCAN-V1 is preferentially localized in the tumor matrix and endothelium, which promotes angiogenesis and tumor development (29).

In this study, we studied the role of ST6GalNAc-I in the modulation of NECTIN2/MUC5AC/VCAN for immune evasion and tumor angiogenesis during LUAD development.

## Results

### Overexpression of ST6GalNAc-I in LUAD.

**Figure 1:**
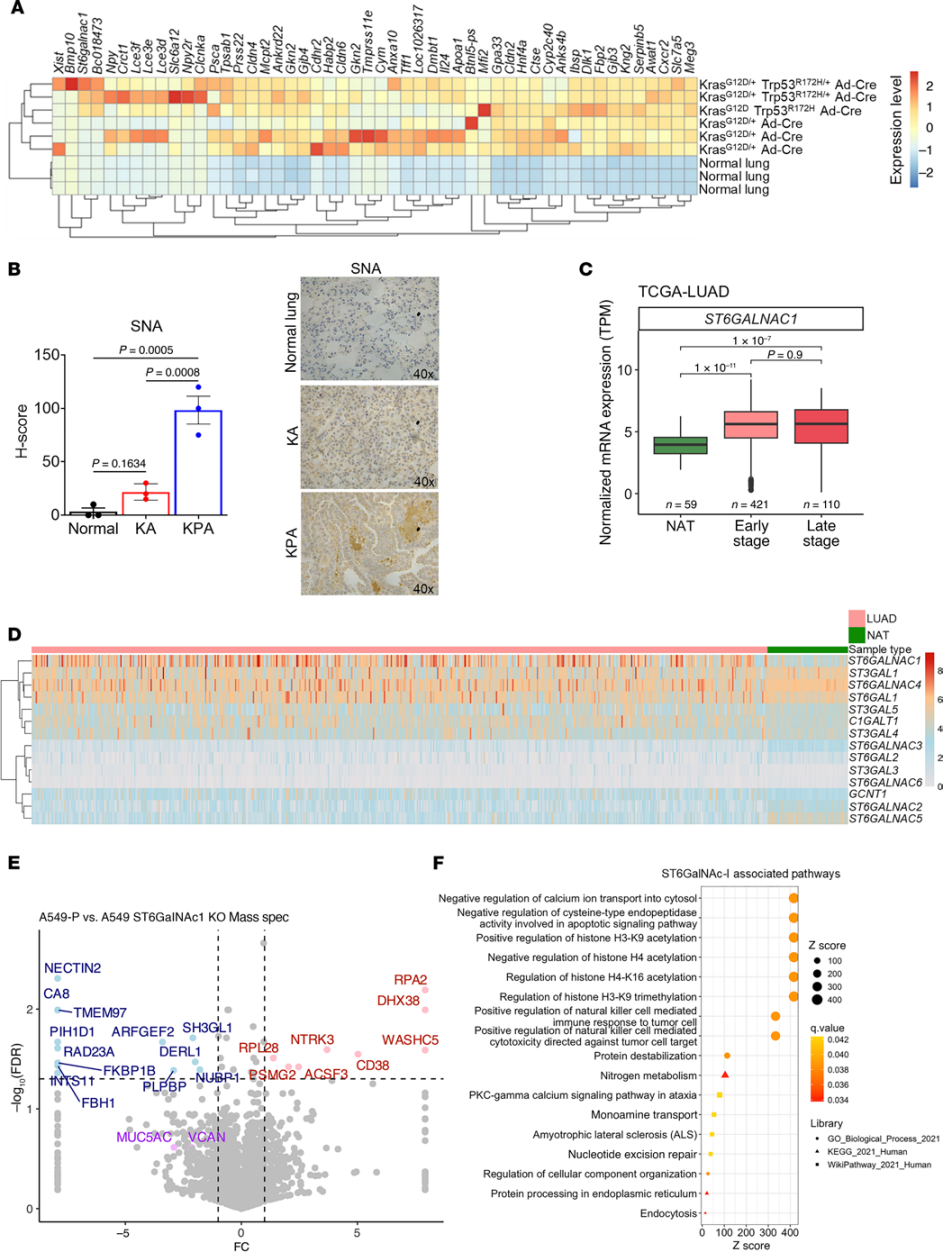
Elevated expression of ST6GalNAc-I in LUAD and its oncogenic role in LUAD. (**A**) The heatmap shows that ST6GalNAc-I is significantly overexpressed in genetically engineered LUAD KPA (*Kras^G12D/+^ Trp53^R172/+^* *Ad-Cre*) compared with KA (*Kras^G12D/+^ Ad-Cre*) lung tumors and normal mouse lung. (**B**) Quantification and representative images of immunohistochemistry of SNA lectin expression in normal, KA, and KPA mouse lung tumor tissues. Significance was determined by 1-way ANOVA (*n* = 3). Original magnification, ×40. (**C**) TCGA dataset shows that *ST6GalNAc-I* is highly overexpressed in early-stage (*n* = 421) and late-stage (*n* = 110) LUAD compared with normal tissue adjacent to the tumor (NAT) (*n* = 59). (**D**) ST6GalNAc-I is the top differentially overexpressed sialyltransferase in LUAD compared with other sialyltransferases or glycosyltransferases, which suggests that targeting ST6GalNAc-I may prevent tumor sialylation–mediated LUAD development. (**E**) The volcano plot represents the mass spectrometry–based proteomic analysis of A549 control versus ST6GalNAc-I–KO cells. NECTIN2 was significantly downregulated in ST6GalNAc-I–KO cells. Red represents significantly upregulated proteins [FDR < 0.05 and log_2_(fold-change) ≥ 1], and blue represents significantly downregulated proteins [FDR < 0.05 and log_2_(fold-change) ≤ 1]. (**F**) Gene Ontology–based pathway analysis using A549 ST6GalNAc-I–KO cells showed NK cell–mediated immune response, apoptosis signaling, protein stability, and metabolic pathways, suggesting that these pathways are associated with ST6GalNAc-I in LUAD.

RNA sequence analysis was performed using genetically engineered LUAD mouse model tumors (*Kras^G12D/+^ Trp53^R172H/+^ Ad-Cre* [KPA] and *Kras^G12D/+^Ad-Cre* [KA]) (15). In comparison with normal lung tissues, several genes were significantly overexpressed in KPA- and KA-derived LUAD tumors, including *Meg3*, *Slc7a5*, *Cxcr1*, *Awat1*, *Kng2*, *Gjb3*, *Mfi2*, and *Dlk1* (Figure 1A). More specifically, *St6galnac-I* was significantly (*P* < 0.001) overexpressed (51.2-fold) in aggressive-type KPA tumor tissues compared with KA and normal lung tissues (Figure 1A). ST6GalNAc-I transfers sialic acid with an α2,6 linkage to GalNAc glycans, generating tumor-related STn (6). We performed *Sambucus nigra* (SNA) lectin staining in KPA and KA tumors to examine the sialylated glycans. SNA binds preferentially to sialic acid attached to terminal galactose in α2,6 linkage and, to a lesser degree, α2,3 linkage (37). Immunohistochemical analysis showed increased α2,6-linked glycans in highly aggressive-type KPA tumors (*P* = 0.0005) (Figure 1B). The Cancer Genome Atlas (TCGA) patient dataset shows significantly higher expression (*P* < 0.001) of *ST6GalNAc-I* in early-stage (*n* = 421) and late-stage (*n* = 110) LUAD samples compared with normal tissue adjacent to the tumor (NAT) (*n* = 59) (Figure 1C). This finding suggests that ST6GalNAc-I is a critical molecule for LUAD development.

### ST6GalNAc-I is a predominant sialyltransferase in LUAD.

In silico analysis of TCGA dataset indicated a relatively high level of *ST6GalNAc-I* expression in LUAD as compared with other isoforms such as *ST6GalNAc-II*, *-III*, *-IV*, and *-V* (Figure 1D). Further, the expression of other sialyltransferases such as *ST3GAL1*, *2*, *3*, *4*, and *5*; *ST6GAL1*; *ST6GAL2*; and *GCNT1* was relatively low compared with *ST6GalNAc-I* (Figure 1D and Supplemental Figure 1A; supplemental material available online with this article; https://doi.org/10.1172/JCI186863DS1). Among all sialyltransferases, *ST6GalNAc-I* was highly overexpressed (*P* = 1.7 × 10^–11^) in tumor tissues but low in NAT (Supplemental Figure 1A). These findings suggest that ST6GalNAc-I is a predominant sialyltransferase for STn synthesis in LUAD. Hence, it is important to understand the biological and functional role of ST6GalNAc-I in LUAD development.

### ST6GalNAc-I–associated glycoproteins and their oncogenic pathways in LUAD.

Mass spectrometry–based (MS-based) proteomic analysis was performed using A549 ST6GalNAc-I–knockout (KO) (*n* = 3) and control cells (*n* = 3). A total of 3,184 proteins were identified and quantified. Protein abundance data were used to calculate FDR values, which were plotted against the mean log_2_(fold change). Proteins with an absolute fold-change (|log_2_FC|) greater than 1 and an FDR value less than 0.05 were considered significantly differentially expressed proteins. We identified that several proteins, including NECTIN2, carbonic anhydrase 8 (CA8), ADP ribosylation factor guanine nucleotide exchange factor 2 (ARFGEF2), cytosolic Fe-S cluster assembly factor NUBP1 (NUBP1), and RAD23 homolog A, nucleotide excision repair protein (RAD23A), were significantly downregulated in A549 ST6GalNAc-I–KO cells (Figure 1E). These molecules are significantly associated with natural killer–mediated immune response to tumor cells, apoptosis, protein stabilization, posttranslational protein modification, and metabolic pathways, suggesting that ST6GalNAc-I mediates these pathways in LUAD (Figure 1F). NECTIN2 has been implicated in tumor progression and metastasis by promotion of an immunosuppressive environment via T and NK cell dysfunction (24, 25). We also observed the decreased expression of MUC5AC in ST6GalNAc-I–KO cells. We previously reported that ST6GalNAc-I modulates MUC5AC for LUAD cell proliferation and migration (15).

Next, we analyzed whether ST6GalNAc-I transcriptionally regulates NECTIN2 and MUC5AC in LUAD. However, we observed that the transcript level of *NECTIN2* and *MUC5AC* was not changed in ST6GalNAc-I–KO cells compared with controls (Supplemental Figure 1B), suggesting that ST6GalNAc-I posttranscriptionally regulates NECTIN2 and MUC5AC in LUAD. As ST6GalNAc-I governs sialylation, its loss of function likely affects the addition of STn to glycoproteins during posttranslational modifications. These sialylation changes may indirectly influence expression, stability, or degradation (38). Hence, to investigate the posttranslational modifications following ST6GalNAc-I depletion in LUAD, we performed inhibition studies on both proteasome- and lysosome-mediated degradation mechanisms. We observed that proteasomal inhibition by MG132 did not rescue the NECTIN2 and MUC5AC levels (Supplemental Figure 1C). However, lysosomal inhibition by hydroxychloroquine (HCQ) rescued NECTIN2 and MUC5AC expression in both control and ST6GalNAc-I–KO cells (Supplemental Figure 1D), suggesting that the loss of sialylation of NECTIN2 and MUC5AC leads to lysosome-mediated degradation. Further, these findings also suggest that ST6GalNAc-I promotes the stability of NECTIN2 and MUC5AC through sialylation in LUAD. A study has demonstrated that loss of α2,6-linked sialic acids is linked with autophagy-mediated degradation (39). Several studies have demonstrated that highly sialylated complex-type glycans contribute to protein stability (40–42).

### NECTIN2 expression and its survival outcomes in LUAD.

**Figure 2:**
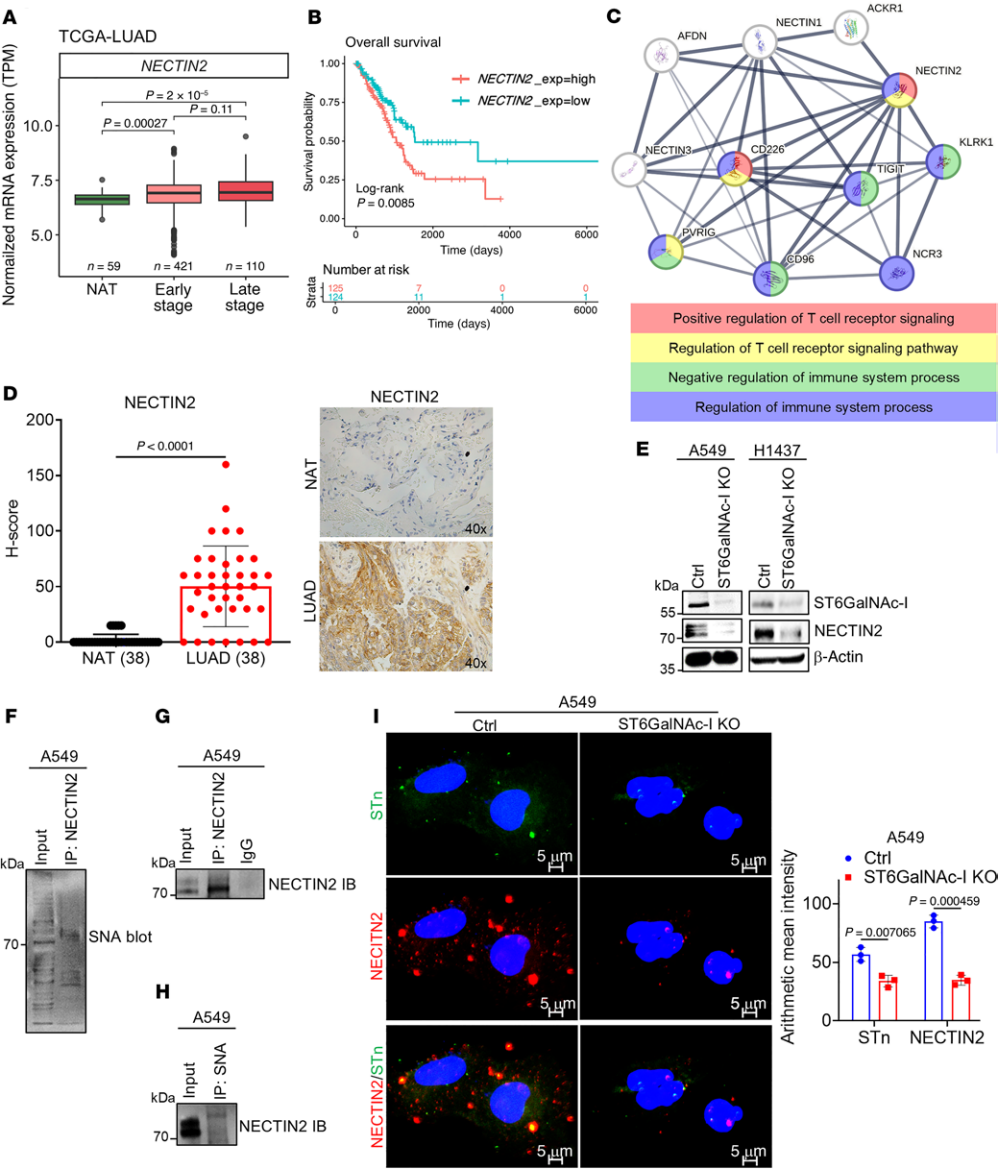
Overexpression of NECTIN2 in LUAD and tumor cell sialylation. (**A** and **B**) In silico analysis indicated that expression of *NECTIN2* is significantly overexpressed and associated with poor survival outcomes in both early- and late-stage LUAD patients. (**C**) Protein-protein interaction networking analysis reveals that tumor cells expressing NECTIN2 induce T cell dysfunction through TIGIT binding, which is associated with immune suppression pathways. (**D**) IHC analysis shows the overexpression of NECTIN2 in LUAD. Data were analyzed using 2-tailed *t* test (*n* = 38). Original magnification, ×10. (**E**) The expression of MUC5AC and NECTIN2 drastically decreased in ST6GalNAc-I–KO and MUC5AC-KD cells (A549 and H1437). (**F** and **G**) Immunoprecipitation assay shows NECTIN2 sialylation in LUAD cells. (**H**) SNA pull-down was performed on A549 cell lysates, followed by immunoblotting with NECTIN2 antibody, suggesting that NECTIN2 carries STn in LUAD cells. (**I**) Immunofluorescence assay reveals the decreased association of NECTIN2 and STn in A549 ST6GalNAc-I–KO cells, and the bar diagram represents the quantification of NECTIN2 and STn using arithmetic mean intensity. Data were analyzed using 2-tailed *t* test (*n* = 3). Scale bars: 5 μm.

Our proteomic analysis identified NECTIN2 as one of the top downregulated molecules in ST6GalNAc-I–KO cells. *NECTIN2* was significantly overexpressed in early-stage (*P* = 0.0002) and late-stage (*P* = 2 × 10^–5^) LUAD compared with NAT (Figure 2A). Higher expression of *NECTIN2* was associated (*P* = 0.008) with poor survival in LUAD (Figure 2B). Moreover, protein-protein interaction network analysis demonstrated that cancer cells expressing NECTIN2 were associated with T cell function through the coinhibitory receptor TIGIT, which promotes T cell dysfunction for immune evasion (Figure 2C). NECTIN2 was strongly associated with T cell receptor signaling and immune suppression–related pathways (Figure 2C). Our immunohistochemical analysis indicated that NECTIN2 was significantly (*P* < 0.0001) overexpressed in LUAD compared with NAT (Figure 2D). Expression of *NECTIN2* was significantly (*P* = 1.65 × 10^–5^) correlated with *ST6GalNAc-I* using LUAD data with the OncoDB tool (https://oncodb.org/) (Supplemental Figure 1E). Further, TCGA-LUAD indicated that *TIGIT* was overexpressed in LUAD compared with NAT (*P* = 2.8 × 10^–9^) (Supplemental Figure 1F). Our immunohistochemical analysis also revealed that TIGIT was overexpressed (*P* < 0.0001) in LUAD-associated lymphocytes (Supplemental Figure 1G). In silico analysis (Gene Expression Omnibus GSE127465) indicated that *NECTIN2* was expressed in tumor cells, whereas *TIGIT* was predominantly expressed in T cells (Supplemental Figure 2A), suggesting that the NECTIN2/TIGIT axis may play a critical role in LUAD development and modulation of antitumor immune response.

### ST6GalNAc-I modulates NECTIN2 sialylation in LUAD.

The UniProt and GlyGen datasets indicate that NECTIN2 has multiple *O*-glycosylation sites, and it has been shown to be involved in tumor development (43, 44). To determine the impact of ST6GalNAc-I on tumor cell sialylation, we used CRISPR/Cas9–based knockout of ST6GalNAc-I in A549 and H1437 cells (Figure 2E). The expression of NECTIN2 was decreased in ST6GalNAc-I–KO cells compared with control cells (Figure 2E). To investigate NECTIN2 sialylation in tumor cells, we performed an SNA lectin pull-down assay in A549 cells. NECTIN2 sialylation was verified by immunoprecipitation of NECTIN2 and SNA lectin blotting (Figure 2F). We also verified the immunoprecipitated NECTIN2 in A549 cells (Figure 2G). Similarly, STn-carrying glycoproteins were pulled down by SNA lectin and probed with NECTIN2 antibody (Figure 2H). Similarly, the confocal analysis also indicated that NECTIN2-associated sialylation was significantly decreased in ST6GalNAc-I–KO cells (*P* = 0.0004) (Figure 2I). These findings indicated that NECTIN2 carries STn in LUAD cells.

### ST6GalNAc-I promotes T cell dysfunction in LUAD.

**Figure 3:**
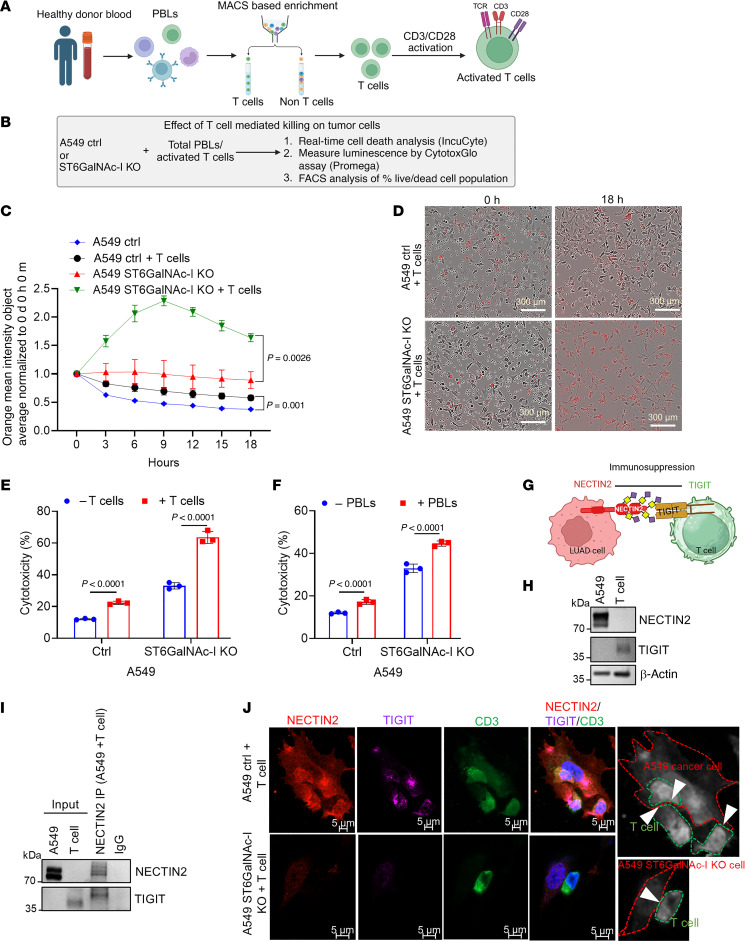
Coculture of A549 ST6GalNAc-I–KO with T cells and its impact on T cell function. (**A** and **B**) Schemes for the isolation of T cells and PBLs from healthy blood donors. A549 ST6GalNAc-I–KO and control cells were cocultured with PBLs and T cell subsets for tumor cell killing assays. (**C** and **D**) We observed that the killing of A549 ST6GalNAc-I–KO cancer cells cocultured with T cells was significantly higher than that of control cells cocultured with T cells as demonstrated by IncuCyte live imaging with CytoTox Red assay. Representative images show differences in red staining indicative of dead cells. Data were analyzed using 2-tailed Student’s *t* test (*n* = 3). Scale bars: 300 μm. (**E** and **F**) Further, coculture of A549 ST6GalNAc-I–KO and control with specific T cells or PBLs also showed increased killing ability of ST6GalNAc-I–KO (48 hours) as indicated by CytoTox-Glo assays. Data were analyzed using 2-tailed Student’s *t* test (*n* = 3). (**G**) The schematic illustrates how tumor cell–expressed NECTIN2 induces T cell dysfunction through the TIGIT receptor. (**H**) Western blot analysis showing that NECTIN2 is specifically expressed in A549 while TIGIT is expressed in T cells. (**I**) Immunoblot of TIGIT and NECTIN2 shows their interaction. The coculture lysates derived from A549 plus T cells were immunoprecipitated with NECTIN2 and probed with both antibodies. (**J**) Immunofluorescence images show the colocalization of NECTIN2 and TIGIT with T cell–specific marker CD3. NECTIN2-TIGIT interacting region (shown by white arrowheads) is represented using black-and-white image. Cancer cells are represented by red dashed lines, and T cells are represented by green dashed lines. Scale bars: 5 μm.

The function of NECTIN2 has been implicated in T cell dysfunction that leads to an immunosuppression environment during tumor development (25, 26, 45). To understand this, we performed a T cell–mediated tumor cell killing assay using the ST6GalNAc-I–KO or control cells with peripheral blood lymphocytes (PBLs) or activated T cells (Figure 3, A and B). The T cells enriched from PBLs using MACS technology were added to the tumor cells at a 1:10 ratio, and the cytotoxicity analysis was performed by the IncuCyte live*-*cell analysis system. We observed higher cell death (*P* = 0.002) in A549 ST6GalNAc-I–KO cells than in control cells, as seen by an increase in orange mean intensity (mean intensity 2.28 vs. 0.99 after 9 hours) (Figure 3, C and D). We observed a similar trend of tumor cell killing with PBLs (*P* < 0.001) (Supplemental Figure 2B). Similarly, we determined the percentage of dead cells by flow cytometry after incubating about 1 × 10^6^ A549 control and ST6GalNAc-I–KO cells with 1 × 10^7^ PBLs and enriched T cells for 48 hours. The live/dead analysis on tumor cells revealed an increase in the percentage of 7-aminoactinomycin D–stained (7-AAD–stained) (dead) cells by 15% for ST6GalNAc-I–KO cells compared with 2% for control cells (Supplemental Figure 2, C and D). Furthermore, the CytoTox-Glo cytotoxicity assay data also showed a significant (*P* < 0.0001) increase in the cell death of ST6GalNAc-I–KO cells incubated with PBLs or T cells (Figure 3, E and F). Our findings suggest that ST6GalNAc-I induces NECTIN2 sialylation in LUAD cells, which leads to T cell dysfunction to promote an immunosuppressive environment (Figure 3G).

Our expression analysis indicated that NECTIN2 was expressed in A549 cancer cells but not in T cells (Figure 3H). On the other hand, expression of TIGIT was detected only in T cells but not in cancer cells (Figure 3H). To find NECTIN2 and TIGIT interaction, NECTIN2 was immunoprecipitated from the coculture lysates (A549 plus T cells) and probed with both TIGIT antibodies followed by NECTIN2 (Figure 3I). We observed that NECTIN2 physically interacted with TIGIT (Figure 3I). In addition, we observed colocalization of NECTIN2 and TIGIT with the T cell–specific marker CD3 (Figure 3J). These findings suggest that cancer cells expressing NECTIN2 interact with T cells through TIGIT, resulting in immunosuppression.

### ST6GalNAc-I–mediated immunosuppression through the NECTIN2/TIGIT axis.

**Figure 4:**
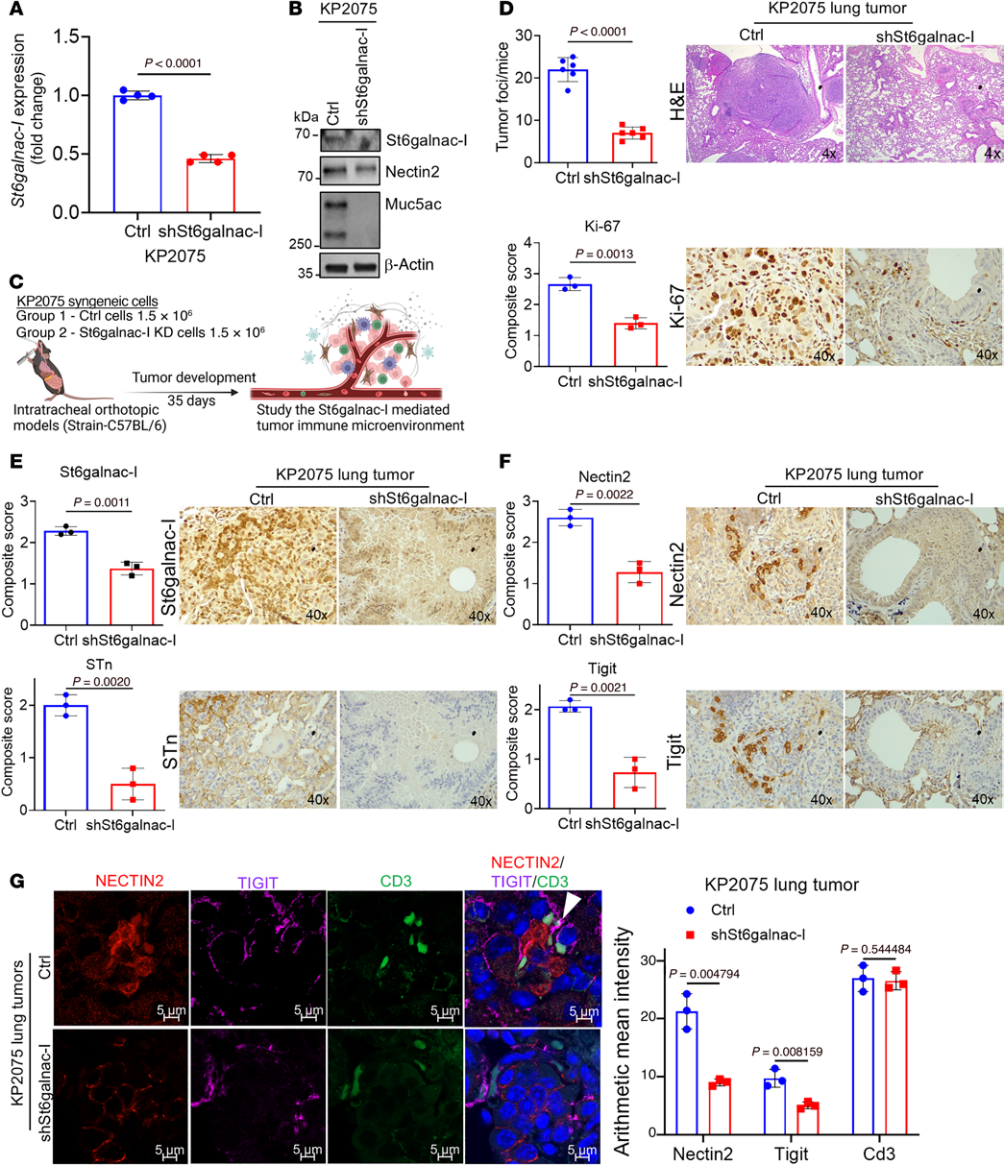
St6galnac-I–associated immunosuppression in LUAD. (**A**) We developed stable mouse St6galnac-I knockdown in mouse syngeneic KP2075 cells. Quantitative real-time PCR analysis shows that the transcript level of *St6galnac-I* was significantly decreased in St6galnac-I–KD cells. Significance was determined by 2-tailed *t* test (*n* = 4). (**B**) Immunoblot shows that Nectin2 and Muc5ac were decreased upon St6galnac-I knockdown. (**C**) Schemes for the intratracheal orthotopic models. (**D**–**F**) Mice injected with St6galnac-I–KD cells showed reduced lung tumor incidence in H&E (*n* = 6) along with decreased Ki-67, St6galnac-I, STn, Nectin2, and Tigit. Significance was determined by 2-tailed *t* test (*n* = 3). Original magnification, ×4 for H&E and ×40 for IHC. (**G**) Left: Immunofluorescence assays indicate decreased association of Nectin2 and Tigit in St6galnac-I–KD tumors. Right: Quantification of the arithmetic mean intensity value of CD3 (green), NECTIN2 (red), and TIGIT (purple) per field of view (*n* = 3). Scale bars: 5 μm.

To understand the ST6GalNAc-I–mediated immunosuppressive environment and tumor development, we performed intratracheal lung orthotopic experiments using St6galnac-I knockdown (KP2075–shSt6galnac-I) in mouse syngeneic cells derived from KPA models in C57BL/6 background (15). St6galnac-I stable knockdown (KD) in KP2075 cells was verified at the transcript and protein levels (Figure 4, A and B). Further, KP2075–shSt6galnac-I cells also showed a decreased expression of Nectin2 and Muc5ac (Figure 4B). Using the intratracheal implantation model (Figure 4C), we observed a significantly decreased lung tumor incidence along with decreased proliferation marker Ki-67 (Figure 4D), St6galnac-I, and STn (Figure 4E). We also observed decreased expression of Nectin2 and Tigit in St6galnac-I–KD tumors (Figure 4F). Confocal images also showed reduced association of Nectin2 and Tigit in St6galnac-I KD–derived xenografts (Figure 4G), suggesting that St6galnac-I plays a critical role in immunosuppression by modulating T cell function through Nectin2.

### ST6GalNAc-I regulates MUC5AC/VCAN in LUAD.

**Figure 5:**
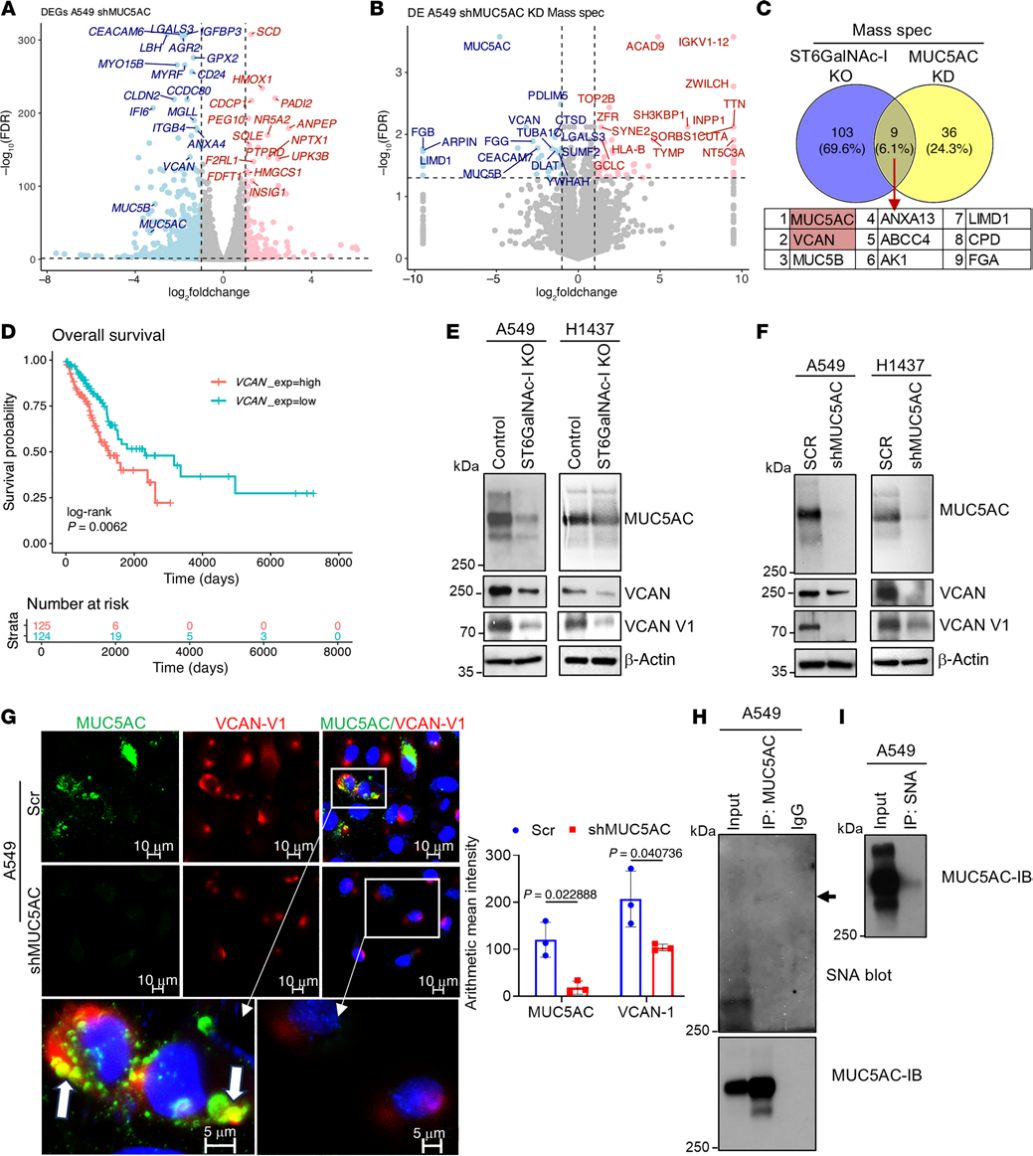
VCAN is the common target of ST6GalNAc-I/MUC5AC in LUAD. (**A** and **B**) Volcano plots for the transcriptomic data (RNA-Seq) [FDR < 0.05 and log_2_(fold change) ≤ 1] and proteomic data [FDR < 0.05 and log_2_(fold change) ≤ 1] reveal that MUC5AC regulates VCAN in LUAD. Red represents significantly upregulated genes/proteins, and blue represents significantly downregulated genes/proteins. (**C**) VCAN is a common downstream molecule of MUC5AC and ST6GalNAc-I as demonstrated by a Venn diagram using MS data (both ST6GalNAc-I–KO and MUC5AC-KD). (**D**) Overall survival analysis indicated that the high expression of *VCAN* showed a poor prognosis in LUAD. (**E** and **F**) Immunoblots show that VCAN and its isoform VCAN-V1 are drastically decreased along with MUC5AC in ST6GalNAc-I–KO and MUC5AC-KD cells (A549 and H1437). (**G**) Confocal images show that MUC5AC and proteoglycan VCAN-V1 are strongly colocalized in the extracellular tumor matrix region of MUC5AC and VCAN-V1 in LUAD cells. Scale bars: 5 μm and 10 μm. Significance was determined by 2-tailed *t* test (*n* = 3). Scr, scramble. (**H**) Immunoprecipitation of MUC5AC in A549 cell lysates, followed by blotting with SNA lectin. The arrow shows SNA staining in the MUC5AC region. (**I**) SNA pull-down in A549 cell lysates and blotting with MUC5AC, suggesting that MUC5AC carries STn in LUAD cells.

Next, we wanted to analyze the mechanism of ST6GalNAc-I–mediated tumor progression and metastasis. We have previously shown that ST6GalNAc-I modulates MUC5AC sialylation, leading to tumor progression (15). MUC5AC is involved in tumor angiogenesis and metastasis in various models (15, 20, 46–49). *MUC5AC* was significantly overexpressed in early- and late-stage LUAD compared with NAT (Supplemental Figure 3A). To investigate the ST6GalNAc-I/MUC5AC–mediated signaling pathways in LUAD, we performed transcriptomic and proteomic analyses using MUC5AC-KD and scramble cells. Transcriptomic (RNA-Seq) (FDR < 0.05) (Figure 5A) and proteomic (MS) (FDR < 0.05) (Figure 5B) data revealed that VCAN was one of the top downregulated molecules in MUC5AC-depleted LUAD cells. Notably, VCAN was also a commonly downregulated molecule of ST6GalNAc-I–KO and MUC5AC-KD cells (Figure 5C), suggesting that the ST6GalNAc-I/MUC5AC axis regulates VCAN expression in LUAD. TCGA data also suggested that *VCAN* was significantly overexpressed in early-stage (*P* = 2.1 × 10^–11^) and late-stage (*P* = 2.2 × 10^–10^) LUAD compared with NAT (Supplemental Figure 3B). Furthermore, higher expression of VCAN was significantly (*P* = 0.006) associated with poor survival of patients with LUAD (Figure 5D). The clinical relevance of the other commonly downregulated molecules of ST6GalNAc-I/MUC5AC is shown in Supplemental Figure 3, C–I.

### ST6GalNAc-I/MUC5AC regulates VCAN isoform V1 in LUAD.

VCAN-V1 and its proteolytic fragment versikine are present in tumor matrix regions and involved in angiogenesis and metastasis of various cancers (31, 34–36). VCAN and its V1 isoform VCAN-V1 (75 kDa) were decreased in both ST6GalNAc-I–depleted (Figure 5E) and MUC5AC-depleted (Figure 5F) cells as compared with controls. On confocal analysis, MUC5AC was strongly colocalized with VCAN-V1 in A549-scramble cells in the tumor matrix region, but this localization was completely abrogated in MUC5AC-KD cells (Figure 5G), suggesting that MUC5AC modulates VCAN-V1 for tumor matrix remodeling during angiogenesis and tumor development. Next, we wanted to analyze the MUC5AC sialylation in LUAD cells. For this experiment, MUC5AC was immunoprecipitated using A549 cell lysates and blotted with SNA lectin (Figure 5H). We perfomed pull-down of STn containing glycoproteins using SNA lectin and blotted with MUC5AC antibody (Figure 5I). These findings indicate that MUC5AC carries STn in LUAD cells.

### Effect of ST6GalNAc-I/MUC5AC on endothelial cell proliferation and migration.

**Figure 6:**
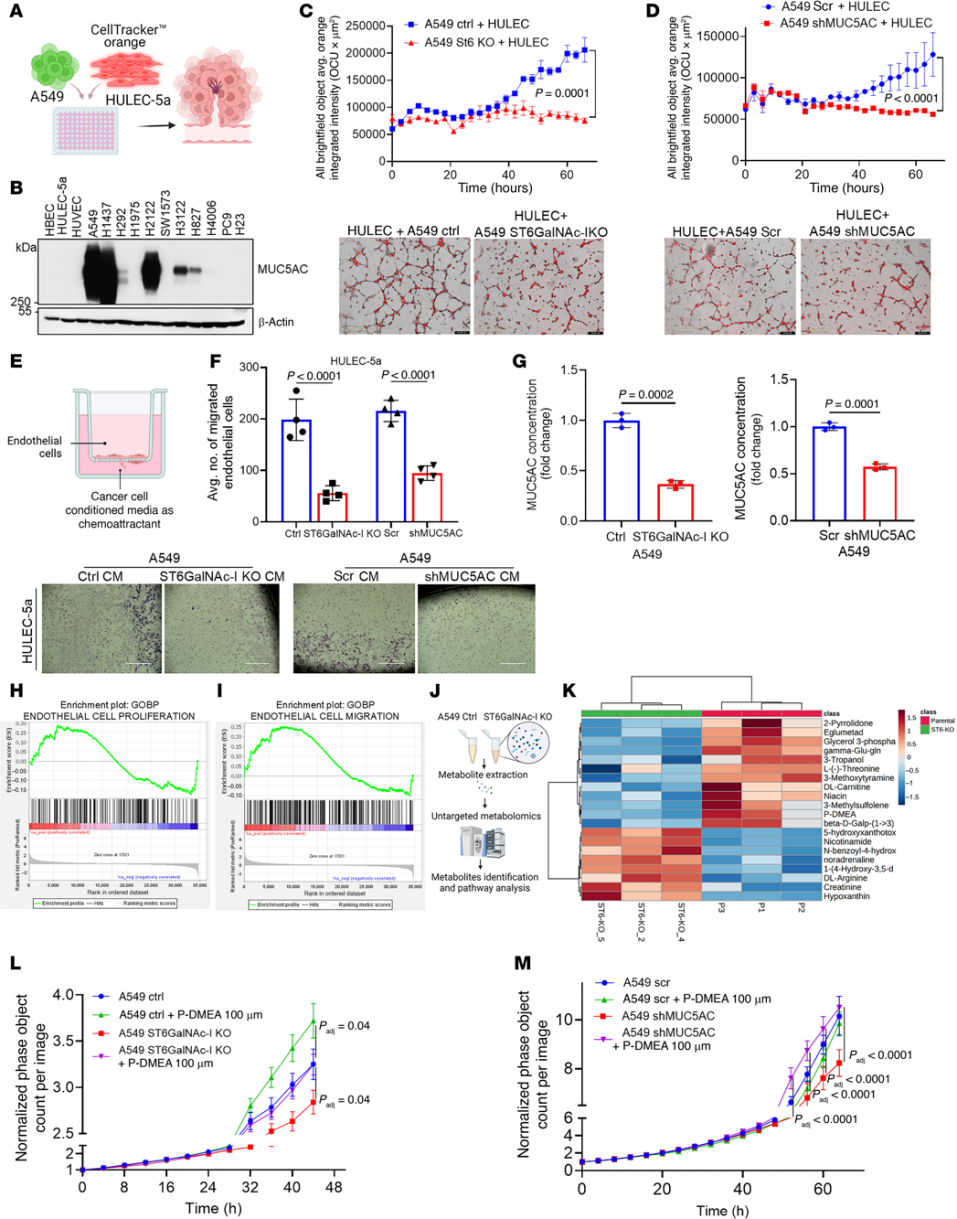
ST6GalNAc-I and MUC5AC promote proliferation and endothelial cell migration. (**A**) Schematic showing coculture for the angiogenesis experiments. (**B**) Western blot analysis indicated expression of MUC5AC in various LUAD cells but not in endothelial cells. (**C** and **D**) IncuCyte-based proliferation and tube formation ability of HULEC-5a cells (labeled with CellTracker Orange CMTMR Dye) cocultured with A549 ST6GalNAc-I–KO or A549 MUC5AC-KD cells in Matrigel-coated plates. ST6GalNAc-I–KO and A549 MUC5AC-KD cells showed poor tube formation ability and proliferation. Significance was determined by 2-tailed *t* test (*n* = 3). Scale bars: 400 μm. (**E**) Schematic showing experimental design for migration assay. (**F**) Transwell migration assay of HULEC-5a cells with A549 ST6GalNAc-I–KO and MUC5AC-KD cell–derived conditioned medium and respective control cells. Data were analyzed using 2-tailed *t* test. Scale bars: 400 μm. (**G**) MUC5AC sandwich ELISA in secretome (conditioned media) of A549 ST6GalNAc-I–KO and A549 MUC5AC-KD cells. Significance was determined by 2-tailed *t* test (*n* = 3). (**H** and **I**) GSEA was done in pre-ranked differentially expressed genes of MUC5AC-KD versus scramble. MUC5AC-KD genes were significantly associated with endothelial cell proliferation and migration. (**J**) Metabolomic analysis was performed using A549 ST6GalNAc-I–KO and control cells. (**K**) Heatmap represents differential expression of metabolites between A549 ST6GalNAc-I–KO and control cells using MetaboAnalyst 6.0. (**L**) P-DMEA–treated (100 μM) ST6GalNAc-I–KO (endogenously low P-DMEA) cells showed increased proliferation properties, suggesting that P-DMEA may be required for LUAD cell growth. Statistical significance was determined by linear mixed model (LMM) (*n* = 3) at 44 hours (*P*_adj_ = 0.04). (**M**) P-DMEA–treated shMUC5AC cells showed significantly increased proliferation after 40–64 hours (*P*_adj_ < 0.01), suggesting that P-DMEA may regulate LUAD cell growth through MUC5AC. Significance was determined by LMM (*n* = 8). *P*_adj_, *P* value adjusted with the Westfall stepdown method.

To investigate the role of ST6GalNAc-I/MUC5AC in endothelial cell function, we cocultured human lung endothelial cells (HULEC-5a) with A549 ST6GalNAc-I–KO or MUC5AC-KD cells, as well as their corresponding control cells, in Matrigel-coated plates (Figure 6A). Our immunoblot analysis verified that MUC5AC was highly elevated in multiple LUAD cell lines but not expressed in human endothelial cells (HUVECs) or HULEC-5a (Figure 6B). We observed that the proliferation and tube formation ability of HULEC-5a were significantly (*P* < 0.0001) reduced in the presence of A549 ST6GalNAc-I–KO (Figure 6C) or A549 MUC5AC-KD (Figure 6D) cells, suggesting that ST6GalNAc-I/MUC5AC might play a critical role in angiogenesis.

To further explore the role of ST6GalNAc-I and MUC5AC in endothelial cell migration, we performed a Transwell migration assay (Figure 6E). In this assay, 2 × 10^4^ HULEC-5a cells were seeded on the upper chamber, and conditioned media (CM) from ST6GalNAC-I–KO or MUC5AC-KD cells and respective controls were added to the bottom chamber. There was a significant (*P* < 0.0001) reduction in the migration of HULEC-5a cells exposed to ST6GalNAC-I–KO or MUC5AC-KD CM compared with respective controls (Figure 6F), suggesting that ST6GalNAc-I/MUC5AC is required for endothelial cell migration. We quantified the secreted MUC5AC in CM of ST6GalNAc-I/MUC5AC–depleted cells and respective controls by ELISA. MUC5AC secretion was significantly (*P* = 0.0002) reduced in the CM of ST6GalNAc-I/MUC5AC–depleted cells compared with control cells (Figure 6G). These findings suggest that LUAD cell–derived MUC5AC plays a critical role in endothelial cell migration.

### ST6GalNAc-I/MUC5AC is associated with endothelial cell migration, proliferation, and vasculature development pathways in LUAD.

We performed a gene set enrichment analysis (GSEA) with the pre-ranked differentially expressed genes (RNA-Seq) of MUC5AC-KD and scramble cells. We found that MUC5AC KD–associated genes were significantly associated with endothelial cell proliferation (Figure 6H), migration (Figure 6I), and vasculature development (Supplemental Figure 3J). Further, in silico analysis indicated a significant association with the LUAD vascular microenvironment gene signatures (VEGF receptor high, E-cadherin low) in patients expressing high levels of MUC5AC as compared with the group with low expression (VEGFA low, E-cadherin high) (Supplemental Figure 3K) (50). These findings demonstrate that MUC5AC is associated with vascular development by modulating vascular microenvironment factors.

### ST6GalNAc-I modulates the metabolite phosphodimethylethanolamine for LUAD cell proliferation.

Our pathways associated with the proteomic data indicated that ST6GalNAc-I is linked with metabolic pathways in LUAD. Several lines of evidence have demonstrated that metabolic pathways are tightly related to glycosylation in cancer (51–53). Therefore, we investigated ST6GalNAc-I–associated metabolites and their potential role in LUAD aggressiveness. To address this question, we performed an untargeted metabolomic analysis of A549 ST6GalNAc-I–KO and control cells based on ultra-high-performance liquid chromatography (UHPLC)/high-resolution (Orbitrap) mass spectrometry (HRMS) (Figure 6J). Certain metabolites, such as 3-methoxytyramine, glycerol-3-phosphate, eglumetad, dl-carnitine, and phosphodimethylethanolamine (P-DMEA), were significantly reduced in ST6GalNAc-I–KO cells compared with controls (Figure 6K). A list of other deregulated metabolites in A549 ST6GalNAc-I–KO cells is given in Supplemental Table 1. To validate the effect of these metabolites on cancer cell proliferation, we treated A549 ST6GalNAc-I–KO and control cells with P-DMEA and other metabolites to understand the effect of these metabolites on LUAD cell aggressiveness. IncuCyte-based real-time proliferation revealed that P-DMEA promoted the proliferation of A549 control cells (Figure 6L). At the same time, we performed the rescue effects of P-DMEA on ST6GalNAc-I–KO cells, where there was a low proliferative index due to the loss of ST6GalNAc-I. The proliferation of ST6GalNAc-I–KO cells was significantly (*P* value adjusted with the Westfall stepdown method [*P*_adj_] = 0.04) increased upon P-DMEA treatment (Figure 6L), suggesting that P-DMEA may be necessary for ST6GalNAc-I–mediated LUAD cell growth. We observed no significant difference in the effect of other metabolites on cancer cell proliferation (data not shown). Further, we wanted to analyze the impact of P-DMEA on the ST6GalNAc-I target molecules NECTIN2 and MUC5AC. We observed a trend similar to that seen with ST6GalNAc-I knockout; P-DMEA rescued the proliferation (*P*_adj_ < 0.01) of MUC5AC-KD (Figure 6M) but not NECTIN-KD cells (Supplemental Figure 4, A and B), suggesting that P-DMEA promotes LUAD cell proliferation through ST6GalNAc-I/MUC5AC pathways. This finding aligns with previous studies indicating that NECTIN2 primarily promotes tumor growth through an immune-dependent mechanism (54). Further, we showed that the metabolic pathways of P-DMEA were involved in phosphatidylcholine biosynthesis (Supplemental Figure 4, C and D). Phosphatidylcholine is the predominant phospholipid in cell membranes and is required for various signaling, including cell proliferation and immune suppression during tumor development (55, 56). Accumulation of phosphatidylcholine is positively correlated with aggressive cancer phenotype (57, 58). Phosphatidylcholine and its lipid mediators are associated with immunosuppression during tumor development (59). These findings suggest that ST6GalNAc-I–mediated P-DMEA/phosphatidylcholine metabolic pathways might be required for the aggressiveness of LUAD. As P-DMEA is an intermediate metabolite in phosphatidylcholine synthesis, ST6GalNAc-I may regulate phosphatidylcholine synthesis through MUC5AC. However, this hypothesis requires further substantiation.

### MUC5AC interacts with the Golgi-associated molecule PRRC1 in LUAD.

**Figure 7:**
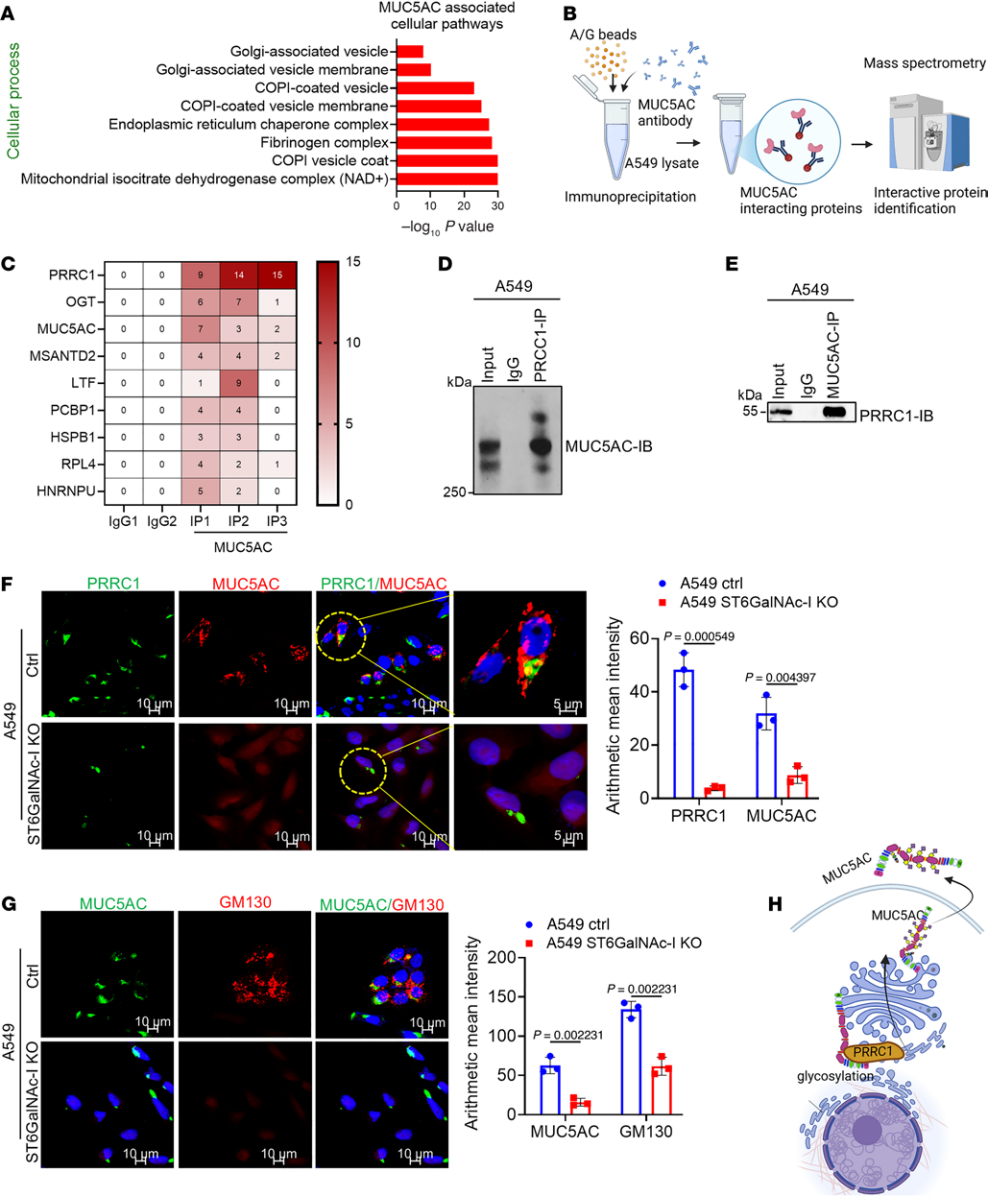
MUC5AC interacts with PRRC1 in LUAD. (**A**) MS-based cellular pathway analysis indicated that MUC5AC is associated with Golgi-associated vesicle and COPI-coated vesicle processes. (**B**) Experimental design for MS-based interactome studies. MS analysis was performed using A549 MUC5AC-KD and scramble cells. (**C**) MS-based MUC5AC interactome studies revealed that MUC5AC strongly interacts with PRRC1 as represented by heatmap. (**D** and **E**) Reciprocal immunoprecipitation assays also revealed that MUC5AC strongly interacts with PRRC1 in LUAD cells. (**F** and **G**) Further, confocal images show that MUC5AC and PRRC1 were strongly colocalized in the Golgi region along with the Golgi-specific marker GM130. Significance was determined by 2-tailed *t* test (*n* = 3). Scale bars: 5 μm and 10 μm. (**H**) Schematic diagram shows the MUC5AC glycosylation process in LUAD cells.

Gene Ontology–based cellular pathway analysis indicated a robust association of MUC5AC with ER-Golgi transport and COPI and COPII vesicle coat formation pathways in LUAD (Figure 7A). Further, the biological pathways analysis indicated that MUC5AC is associated with heterotypic cell-cell adhesion and peptide secretion pathways (Supplemental Figure 5, A and B). This finding raises the possibility that MUC5AC might have a crucial role in ER-Golgi vesicle transport during LUAD development. To understand the mechanism of ST6GalNAc-I–mediated MUC5AC glycosylation, we performed MS analysis using A549 cells (Figure 7B). Our MS-based interactome studies identified PRRC1 as one of the top interacting molecules with MUC5AC (Figure 7C). To validate the MUC5AC-interacting molecules, our reciprocal immunoprecipitation assays indicated that MUC5AC strongly interacted with PRRC1 in LUAD cells (Figure 7, D and E). Further, the interaction of MUC5AC and PRRC1 was abrogated in ST6GalNAc-I–KO cells (Figure 7F). The localization of MUC5AC and PRRC1 at the Golgi region was further verified using the Golgi marker GM130 (Figure 7G). PRRC1 is preferentially localized in the ER exit sites; it has been shown to regulate membrane association of the COPII coat and facilitates ER-to-Golgi trafficking (23). These findings suggest that ST6GalNAc-I regulates the interaction between MUC5AC and PRRC1 in LUAD cells, a process that may be essential for the transport of MUC5AC from the ER to the Golgi during glycosylation (Figure 7H).

### ST6GalNAc-I– and MUC5AC–deficient tumors showed reduced tumor incidence, tumor angiogenesis, and liver metastasis.

**Figure 8:**
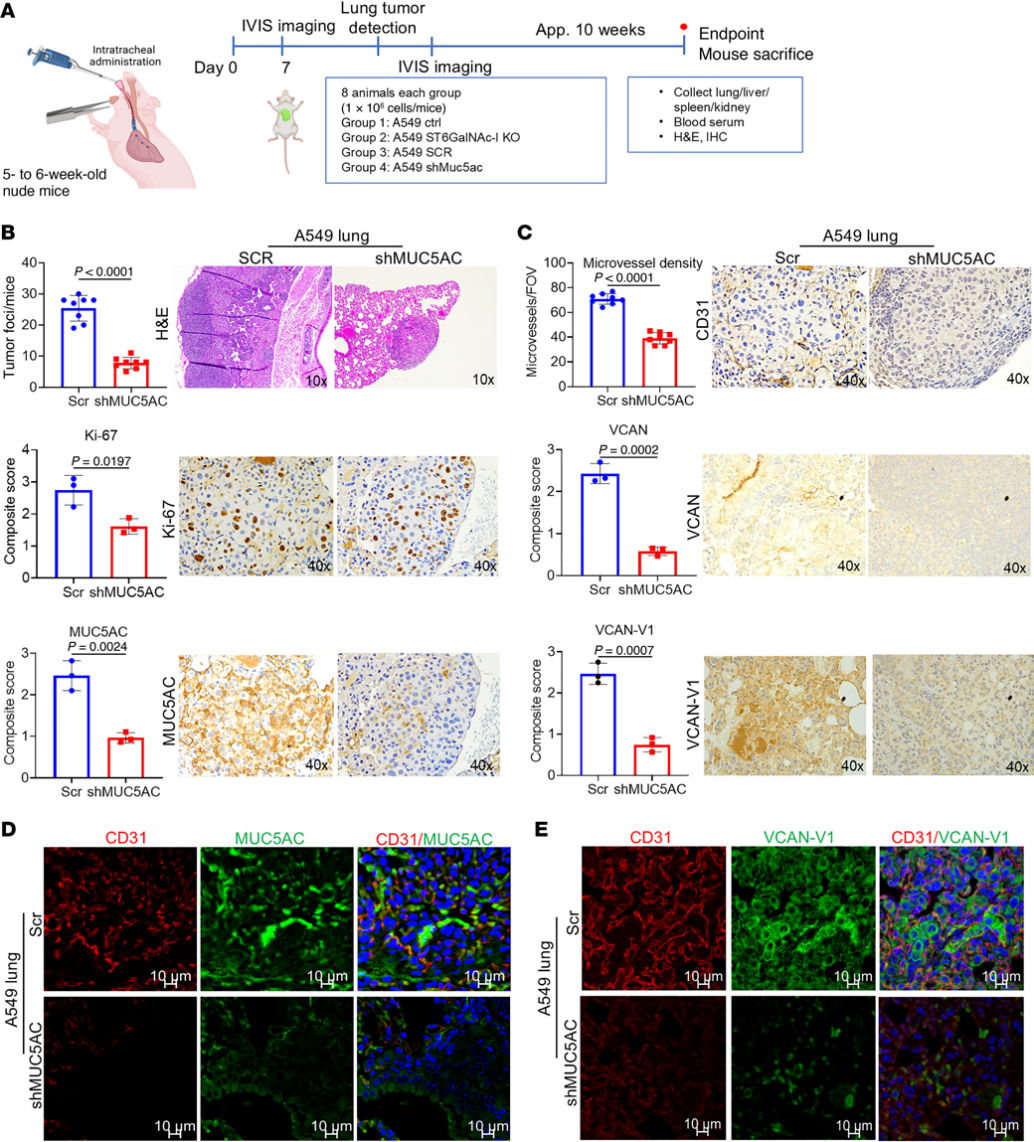
MUC5AC is associated with angiogenesis and tumor development. (**A**) Scheme of in vivo intratracheal models using human non–small cell lung cancer cells in athymic nude mice. (**B**) Mice injected with A549 MUC5AC-KD cells had significantly reduced lung tumor foci compared with mice injected with respective control cells (*n* = 8). (**B** and **C**) IHC shows that the expression of Ki-67, MUC5AC, CD31, VCAN, and VCAN-V1 was significantly reduced in MUC5AC-KD tumors. Original magnification, ×10 for H&E and ×40 for IHC. Significance was determined by 2-tailed *t* test (*n* = 3). (**C**, top panel) The microvessel density was quantified using CD31-stained tumor sections in 8 fields for each tumor section and presented as a mean number per field of view (FOV; 0.2 mm^2^). Significance was determined by 2-tailed *t* test (*n* = 3). (**D** and **E**) Confocal images show decreased colocalization of CD31 with MUC5AC and VCAN-V1 in MUC5AC KD–derived xenograft. Scale bars: 10 μm.

To determine the role of ST6GalNAc-I and MUC5AC in non–small cell lung cancer development and metastasis, we performed intratracheal lung orthotopic experiments using A549 ST6GalNAc-I–KO and MUC5AC-KD and respective control cells (Figure 8A). ST6GalNAc-I–KO and MUC5AC-KD tumors showed reduced lung tumor incidence and decreased expression of the proliferative marker Ki-67, ST6GalNAc-I, and MUC5AC (Figure 8B and Supplemental Figure 6A). In addition, to verify the α2,6-linked glycan in LUAD, we performed immunostaining of SNA in the lung xenografts derived from ST6GalNAc-I–KO and controls. We observed significantly decreased α2,6-linked glycans in ST6GalNAc-I KO–derived tumors (*P* = 0.01) (Supplemental Figure 6A, bottom panel). Further, the expression of CD31, VCAN, VCAN-V1, and NECTIN2 was significantly decreased in ST6GalNAc-I KO– and MUC5AC KD–derived tumors (Figure 8C and Supplemental Figure 6B). Microvessel density was also significantly reduced in ST6GalNAc-I KO– and MUC5AC KD–derived tumors (Figure 8C and Supplemental Figure 6B). These findings indicate that ST6GalNAc-I and MUC5AC modulate tumor angiogenesis. Further, co-occurrence of MUC5AC/CD31 or VCAN-V1/CD31 was drastically decreased in MUC5AC KD–derived (Figure 8, D and E, and Supplemental Figure 7, A and B, quantification) or ST6GalNAc-I KO–derived (Supplemental Figure 6C) tumor xenograft tissues. Similarly, enriched coexpression of MUC5AC/CD31 and CD31/VCAN-V1 in human and mouse LUAD tissues (Supplemental Figure 7, C and D) suggests that MUC5AC/VCAN-V1 plays a critical role in tumor angiogenesis. Similarly, St6galnac-I–KD mouse tumors also showed reduced Muc5ac and Vcan-V1, along with decreased Cd31-based microvessel density (Supplemental Figure 8). These findings suggest that ST6GalNAc-I/MUC5AC is a critical pathway for tumor angiogenesis in LUAD.

We also observed that the incidence of liver metastasis was significantly reduced in mice injected with ST6GalNAc-I–KO (*P* = 0.0005) and MUC5AC-KD (*P* = 0/008) cells (Supplemental Figure 9, A and B). In addition, trichrome staining data also revealed that tumor matrix was reduced in A549 ST6GalNAc-I KO–derived (*P* = 0.002) and A549 MUC5AC KD–derived tumor xenograft tissues (*P* = 0.0008) compared with their respective controls (Supplemental Figure 9, C and D). Overall, these findings suggest that ST6GalNAc-I induced MUC5AC sialylation for LUAD growth and liver metastasis through altering VCAN-V1.

## Discussion

Tumor immunosuppressive environment promotes disease aggressiveness and therapy resistance in various cancers (37, 60–62). We have previously described that ST6GalNAc-I is overexpressed in lung cancer (15). The function of glycoproteins is determined by glycan residues, and aberrant glycosylation leads to immune suppression, tumor progression, and metastasis (18, 19). Several lines of evidence have demonstrated that sialylated glycans play a critical role in immune evasion and metastasis (37, 63, 64). In this study, we identified that ST6GalNAc-I induces the sialylation of NECTIN2 and MUC5AC in LUAD cells. Our findings also demonstrate that loss of ST6GalNAc-I–associated NECTIN2 and MUC5AC sialylation leads to lysosome-mediated degradation in LUAD, suggesting that ST6GalNAc-I promotes the stability of NECTIN2 and MUC5AC through sialylation. Previous studies have demonstrated that glycosylated proteins have increased stability in various cancer cells (38, 39).

A recent study has demonstrated that cancer cells expressing NECTIN2 promote an immunosuppressive environment through T cell dysfunction (26). We showed that ST6GalNAc-I–deficient cells were effectively killed by T cells in the coculture experiment conditions. Our findings from the MS-based annotations and in vitro and in vivo evidence suggest that ST6GalNAc-I mediates T cell dysfunction through NECTIN2 sialylation. Additionally, St6galnac-I–deficient mouse LUAD tumors exhibited reduced association of Nectin2 with Tigit, indicating that St6galnac-I contributes to tumor immunosuppressive environment through T cell dysfunction mediated by Nectin2. It has been reported that targeting the NECTIN2/TIGIT axis along with PD-L1 blockage increased the survival of mice bearing neuroblastoma tumors (26), suggesting that the NECTIN2/TIGIT axis is a critical immune checkpoint in LUAD progression. These findings provide more direct and comprehensive evidence of the ST6GalNAc-I/NECTIN2/TIGIT axis’s role as a glyco-immune checkpoint. Therefore, targeting NECTIN2 sialylation may prevent NECTIN2/TIGIT interaction in LUAD and improve survival outcomes.

Further, increased expression of ST6GalNAc-I leads to augmented STn expression that has been associated with enhanced metastasis through altered mucin signaling (12, 65). Dysregulation of mucin glycosylation contributes to the progression and metastasis of various cancers, including lung cancer (66, 67). We have reported that ST6GalNAc-I is overexpressed in LUAD and associated with angiogenesis and liver metastasis through MUC5AC (15). MUC5AC is overexpressed in LUAD and correlated with poor outcomes (15). In this study, we identified the role of ST6GalNAc-I in tumor angiogenesis and liver metastasis through modulation of the glycoproteins MUC5AC and VCAN-V1. ST6GalNAc-I/MUC5AC–deficient LUAD cells showed decreased expression of VCAN-V1. VCAN-V1 has been implicated in angiogenesis by altering the tumor matrix (29, 32, 36).

Tumor angiogenesis and metastasis are cascades of events in cancer progression mediated by several factors, including growth factors, adhesion factors, ECM proteins, proteases, and transcription factors (68). We showed that MUC5AC and VCAN-V1 are preferentially colocalized in the extracellular matrix region and enriched in the CD31^+^ endothelial cell region. Mechanistically, ST6GalNAc-I/MUC5AC mediates LUAD development through vasculogenesis and metastasis through the proteoglycan VCAN-V1. In addition, we showed that ST6GalNAc-I/MUC5AC mediates lung endothelial cell proliferation and migration, suggesting that the ST6GalNAc-I/MUC5AC axis is required for tumor angiogenesis. The organs most affected by lung cancer metastases are brain, liver, bone, and adrenal glands (69, 70). We identified that ST6GalNAc-I/MUC5AC–deficient LUAD cells display reduced lung tumor incidence and liver metastasis. In support of this finding, we observed reduced ST6GalNAc-I, MUC5AC, and VCAN-V1 in ST6GalNAc-I/MUC5AC–depleted lung tumor tissues, suggesting that the ST6GalNAc-I/MUC5AC axis is required for lung cancer liver metastasis.

The altered metabolism of cancer cells, now recognized as a key hallmark of cancer, is driven by changes in signaling pathways, protein expression, and other molecular mechanisms, along with specific biochemical adaptations during carcinogenesis (71). We identified altered metabolites in ST6GalNAc-I–depleted cells using a UHPLC/high-resolution (Orbitrap) mass spectrometry (HRMS) approach. Specifically, ST6GalNAc-I regulates the metabolite P-DMEA, which is involved in glycerophospholipid metabolism, contributing to the biosynthesis of membrane phosphatidylcholine. The induction of P-DMEA significantly promotes the proliferation of LUAD cells through MUC5AC, suggesting that P-DMEA is necessary for LUAD growth through the ST6GalNAc-I/MUC5AC axis. Studies have demonstrated that reprogramming of glycerophospholipid metabolism is essential in cancer cells to adapt energy utilization and cellular signaling to promote cell survival and the onset of multidrug resistance (72, 73). A recent study reported that P-DMEA is one of the metabolic biomarkers for renal cell carcinoma (74). Additionally, the conversion of phosphatidylethanolamine to phosphatidylcholine by phosphatidylethanolamine *N*-methyltransferase is elevated in glioblastoma tumorigenesis (75). Phosphatidylcholine is the predominant phospholipid in cell membranes that are required for cancer cell growth and immune suppression during tumor development (55, 56).

In addition, ST6GalNAc-I/MUC5AC–associated molecules are strongly linked with Golgi-associated vesicle membrane formation pathways. We identified that PRRC1 strongly interacted with MUC5AC. A study demonstrated that PRRC1 is preferentially localized in ER exit sites and is necessary for ER-Golgi transport of transmembrane and secretory proteins (23). Based on these findings, we propose that PRRC1 may regulate the transport of MUC5AC from ER to Golgi. Further, we believe that PRRC1 may facilitate MUC5AC transport during the glycosylation process. Notably, the interaction between MUC5AC and PRRC1 was abrogated in ST6GalNAc-I–deficient cells, suggesting that ST6GalNAc-I may play a role in MUC5AC vesicle transport through PRRC1.

**Figure 9:**
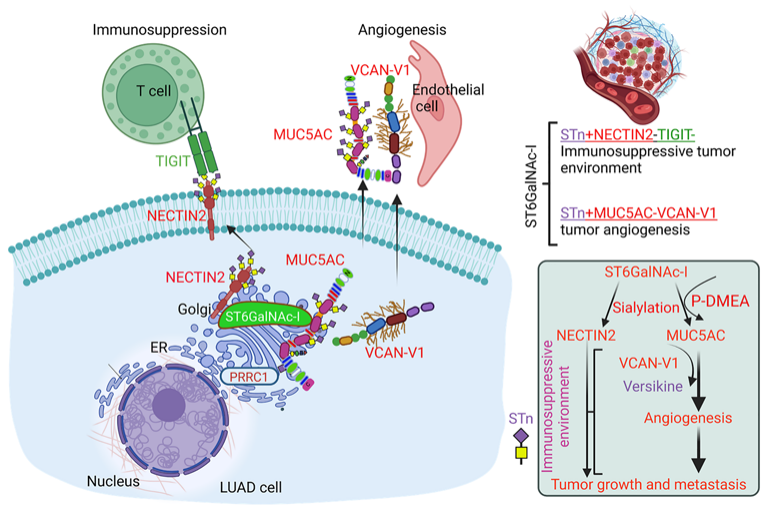
Schematic representation of ST6GalNAc-I–mediated immunosuppression and tumor angiogenesis. Our findings indicated that ST6GalNAc-I induces tumor cell sialylation through NECTIN2 and MUC5AC for immune evasion and tumor angiogenesis. Hence, targeting ST6GalNAc-I or tumor cell sialylation may prevent LUAD development and metastasis. Further targeting ST6GalNAc-I–associated NECTIN2 sialylation may enhance the immune checkpoint inhibitors to improve survival of patients with LUAD.

Overall, our studies demonstrated that ST6GalNAc-I–induced tumor cell sialylation through NECTIN2 and MUC5AC contributes to immune evasion and tumor angiogenesis (Figure 9). These data suggest that strategies to reduce sialylation of NECTIN2 and MUC5AC could improve the survival of LUAD patients and warrant further investigation in LUAD and other malignancies. Our future studies will focus on ST6GalNAc-I–mediated immunosuppression in LUAD using genetically engineered ST6GalNAc-I–knockout mice. The desialylation of tumor cells using the α2,6-glycan–specific inhibitor P-SiaFNEtoc (76, 77) along with immune checkpoint inhibitors could enhance antitumor immunity.

## Methods

### Sex as a biological variable.

Both male and female mice (athymic and C57BL/6) were used in this study. We did not observe any difference between the sexes.

### Cell culture and generation of stable gene knockdown.

Non–small cell lung cancer (NSCLC) cell lines A549, H1437, H2122, H292, H1975, SW1573, H827, H4006, H3122, PC9, and H23; HULEC-5a and HUVEC endothelial cells; and normal human bronchial epithelial cells were purchased from the American Type Culture Collection and maintained in RPMI medium supplemented with 10% FBS and 1× penicillin and streptomycin. Mouse LUAD cells KP2075 were cultured in DMEM with the same supplements (15). The mutational background of LUAD cell lines is listed in Supplemental Table 2. ST6GalNAc-I knockout was performed using the CRISPR/Cas9 method (ST6GalNAc-I CRISPR guide RNA 2 cloned in pSpCas9 BB-2A-GFP [PX458] vector) (15). The guide RNA sequence (GGCCAACCAGGCACCGCCGG) was used for targeting ST6GalNAc-I in multiple NSCLC cells A549, H1437, and H292 (15). Scramble control and pSUPER-Retro-shMUC5AC were transfected into Phoenix cells(American Type Culture Collection) using Lipofectamine 2000 to generate viral particles (Invitrogen) (20, 78, 79) and used to infect A549 and H1437 cells. The pooled population of MUC5AC-KD cells was obtained using puromycin selection (4 μg/mL) and was further expanded to confluent levels to obtain stably transfected cells.

### Immunohistochemistry and immunofluorescence.

Immunohistochemistry was performed in human LUAD tissues from the University of Nebraska Medical Center (UNMC) biobank (*n* = 38), NSCLC cell–derived xenografts, and mouse syngeneic tumors. ST6GalNAc-I (catalog AB229816, Abcam), MUC5AC (CLH2, catalog MAB2011, Millipore), NECTIN2 (catalog 27171-1-AP, ProteinTech), VCAN (catalog MA5-42721, Invitrogen), VCAN-V1 (catalog PA1-1748A, Invitrogen), CD31 (catalog AB222783, Abcam), TIGIT (catalog NBP2-79794, Novus Biologicals), and Ki-67 (catalog AB15580, Abcam) antibodies were used as primary antibodies. ImmPRESS Universal (catalog MP-700, Vector Laboratories) anti-mouse IgG/anti-rabbit IgG was used for secondary antibody incubation, and the sections were developed using DAB, counterstained with hematoxylin, and mounted with PerMount (SP15-100, Thermo Fisher Scientific) (79). Immunostaining was evaluated by a trained pathologist masked to the clinical information. The xenograft tissues were quantitatively assessed by Fiji-ImageJ software (80). Microvessel density was analyzed as described previously (81). Briefly, CD31^+^ microvessels in tumor sections were counted at ×40 original magnification in 8 fields of each section. Results are presented as mean number of microvessels per field of view (0.2 mm^2^) ± standard deviation.

Immunofluorescence studies were conducted on the tissues and cells using MUC5AC (45M1 clone, catalog ab3649, Abcam; dilution 1:100), STn (clone B35.1, catalog LSC170901, LSBio; dilution 1:50), PRRC1 (catalog A305-783A-T, Bethyl Laboratories; dilution 1:1,000), GM130 (catalog 11308-1-AP, ProteinTech), VCAN-V1 (catalog PA1-1748A, Invitrogen; dilution 1:500), CD31 (catalog AB222783, Abcam; dilution 1:100), CD3 (catalog ab11089, Abcam), NECTIN2 (catalog 27171-1-AP, ProteinTech), and TIGIT (catalog NBP2-79794, Novus Biologicals) as primary antibodies. Anti-rat FITC (catalog 112-095-003, Jackson ImmunoResearch Laboratories Inc.), anti-mouse Alexa Fluor 647 (catalog A-21235, Thermo Fisher Scientific), and anti-rabbit Alexa Fluor 568 (catalog A-11004, Thermo Fisher Scientific) were used as secondary antibodies and then imaged using an LSM710 confocal microscope (Carl Zeiss GmbH).

### Immunoprecipitation and MS analysis.

Immunoprecipitation was performed as previously described (69). MUC5AC (CLH2 clone), PRRC1, and IgG antibodies were immunoprecipitated with total lysates (500 μg) isolated from A549 cells using protein A+G Sepharose beads (catalog sc-2003, Santa Cruz Biotechnology). The input, immunoprecipitants, and IgG were electrophoretically resolved and immunoblotted to find interacting proteins.

### Lectin pull-down assay.

Biotinylated *Sambucus nigra* (SNA) (elderberry bark) lectin (catalog B-1305-2, Vector Laboratories), which preferentially binds sialic acid in an α2,6 linkage, was incubated overnight with total lysates (500 μg) isolated from A549 cells. Protein A+G Sepharose beads were added to the lysate-lectin mix and incubated on a rotating platform for 6 hours at 4°C and then washed 4 times with RIPA buffer. The precipitants were electrophoretically resolved on a 2% SDS-agarose gel for MUC5AC and 10% SDS-PAGE for NECTIN2 and SNA. The SNA blots were processed using VECTASTAIN Elite ABC-HRP (catalog PK-6100, Vector Laboratories) and developed.

### Immunoblot.

Total protein was isolated using the RIPA buffer (50 mM Tris-HCl, 150 mM NaCl, 1% NP-40, 0.5% sodium deoxycholate, and 0.1% SDS) containing 1× protease inhibitor cocktail. About 20–40 μg of total protein was resolved in SDS-PAGE or 2% agarose gel for MUC5AC, transferred, and developed as previously described (69). MUC5AC (CLH2, MAB2011, MilliporeSigma; 1:1,000), ST6GalNAc-I (catalog AB229816, Abcam), NECTIN2 (catalog 27171-1-AP, ProteinTech), VCAN (catalog MA5-42721, Invitrogen), VCAN-V1 (catalog PA1-1748A, Invitrogen), PRRC1 (catalog A305-783A-T, Bethyl Laboratories), p53 (sc-126, Santa Cruz Biotechnology), LC3B (83506, Cell Signaling Technology), and anti–β-actin (catalog 4967, Cell Signaling Technology) were used as primary antibodies.

### Lentiviral production, transduction, and selection of stable cell lines.

The lentivirus plasmids, mouse St6galnac1 (catalog VB900061-0626fdu, VectorBuilder Inc.), and human NECTIN2 (catalog VB900058-7167jxc, VectorBuilder Inc.) were transfected into HEK293T cells (American Type Culture Collection) to produce viral particles using the third-generation packaging combination pMDLg/pRRE (12251, Addgene), pRSV-Rev (12253, Addgene), and pMD2.G (12259, Addgene). The transduced cells were sorted by FACS and selected with 2 μg/mL puromycin (ant-pr, InvivoGen).

### Mouse intratracheal lung orthotopic experiments.

Approximately 1 million viable GFP-labeled parental, ST6GalNAc-I–KO, scramble, and MUC5AC-KD cancer cells (A549) in 50 μL PBS were intratracheally injected into the lungs of 5- to 6-week-old athymic nude mice (*n* = 8 per group). The mice were monitored every week for metastasis by In Vivo Imaging System imaging. After 70 days, the mice were sacrificed, and tissues were collected for further investigation (69). To study the immune suppression in vivo, 1.5 × 10^6^ cells (KP2075 control and shSt6galnac-I) were introduced intratracheally into the lungs of 5- to 6-week-old C57BL/6 mice (*n* = 6 per group), and the mice were sacrificed after 35 days to collect lung tumor tissues for further investigations.

### Coculture of NSCLC and endothelial cells for tube formation and proliferation.

GFP-labeled A549 ST6GalNAc-I–KO and A549 MUC5AC-KD (3,000 cells per well) and respective control cells were cocultured with HULEC-5a endothelial cells (labeled with CellTracker Orange CMTMR Dye, Thermo Fisher Scientific) (20,000 cells per well) in Matrigel-coated (Corning Matrigel Basement Membrane Matrix) 96-well plates, and then the proliferation ability of these cells was quantified for different time points using an IncuCyte (Sartorius) live imaging instrument.

### Endothelial cell migration assay.

Transwell migration assay was performed using endothelial cells (HULEC-5a) with culture supernatant of A549 ST6GalNAc-I–KO and A549 MUC5AC-KD cells and respective controls. For this experiment, HULEC-5a cells (2.0 × 10^4^) were seeded on the top chamber of a Boyden chamber (8 μm pore size) well, and serum-deprived conditioned media of A549 ST6GalNAc-I–KO and A549 MUC5AC-KD cells and respective controls were kept in the bottom chamber. After 24 hours, the transmigrated endothelial cells were stained, imaged, and counted using ImageJ software. Secretory levels of MUC5AC in the conditioned media were quantified by sandwich ELISA as described previously (48).

### Isolation of T cells and tumor cell killing assay by coculture.

Blood from healthy volunteer donors was used for the isolation of PBLs (deidentified samples) using density gradient centrifugation (Elutriation Core Facility, UNMC). T cells were isolated and purified from PBLs with autoMACS (Miltenyi Biotec) using the human Pan T Cell Isolation Kit (130-096-535, Miltenyi Biotec) according to the manufacturer’s instructions. Isolated T cells were cultured and activated with CD3/CD28 Dynabeads (Thermo Fisher Scientific) at a 1:2 ratio for 24 hours. A549 ST6GalNAc-I–KO and control cells were cocultured with T cells/PBLs at a 1:10 ratio in a 96-well plate with complete RPMI medium (26). To determine cell death, we added CytoTox dye (Sartorius) to the cocultures, and the plate was read using the IncuCyte live-cell imaging system. In addition, we measured cytotoxicity in 48-hour cocultures using a CytoTox-Glo (catalog G9290, Promega) kit for luminescence. The luminescence signal generated from control cells and T cells was used to normalize the values obtained from the coculture of tumor cells and enriched T cells.

To determine the percentage of dead cells, we incubated 1 × 10^6^ A549 ST6GalNAc-I–KO and control cells with enriched and activated T cells for 24 hours followed by FACS analysis using LIVE/DEAD Fixable Blue Dead Cell Stain Kit (L34962, Invitrogen). At the end of the experiment, the cells were washed with wash buffer (0.5% BSA in PBS) and collected using cell dissociation buffer (stripping buffer) for 10 minutes. The pooled cells were incubated with the LIVE/DEAD stain according to the manufacturer’s protocol. The cells were fixed with 4% paraformaldehyde for FACS, and the percentage of dead cells was calculated.

### NECTIN2-TIGIT interaction.

To determine biochemical interaction, 0.5 × 10^6^ A549 (control and ST6GalNAc-I–KO) cells were cocultured with 5 × 10^6^ activated T cells for 24 hours, and the lysate was collected using RIPA buffer. Immunoprecipitation was carried out using NECTIN2 (catalog 27171-1-AP, ProteinTech) per the abovementioned protocol and detected using TIGIT (catalog NBP2-79794, Novus Biologicals). For immunofluorescence, A549 (control and ST6GalNAc-I–KO) cells were cocultured with activated T cells (1:10 ratio) for 24 hours, fixed, and processed for antibody incubation.

### Proteomic analysis and data processing.

MS-based proteomic analysis was performed with Glycomic Profiling Service (Creative Proteomics). Approximately 100 μg of total protein of A549 ST6GalNAc-I–KO and A549 MUC5AC-KD and respective control cells was used for the MS analysis in triplicates, and the deregulated proteins were analyzed for pathway analysis.

### Metabolite extraction and untargeted metabolomic analysis.

To extract metabolites, A549 ST6GalNAc-I–KO and control cells (2 × 10^6^ cells per 100 mm plate) were seeded and cultured for 48 hours, and the cells were quickly washed with MS-grade water twice and immediately kept on dry ice. One milliliter of chilled 80% methanol containing 1 mM of Canonical Amino Acid Standard Mix (MSK-CAA-1, Cambridge Isotope Laboratory Inc), which contained all 20 proteogenic amino acids labeled with ^13^C and ^15^N, was added to the plates and kept at –80°C for 15 minutes. The cells were scraped and centrifuged at 16,000*g* for 30 minutes at 4°C (82). The supernatants containing metabolites were vacuum-dried in a SpeedVac (Thermo Fisher Scientific) for 4 hours and resuspended in 40 μL of MS-grade acetonitrile/water/isopropanol (50:40:10 vol/vol/vol) according to the protein concentrations for liquid chromatography–MS (LC-MS) analysis. A high-resolution (Orbitrap) mass spectrometer (HRMS) of the Thermo Orbitrap Exploris 480 LC-MS system connected with Thermo Vanquish Neo UHPLC was used for the metabolite profiling at the UNMC Mass Spectrometry Core Facility. The MetaboAnalyst version 6.0 platform was used to process, analyze, and interpret data from untargeted metabolomics (83).

### Bioinformatics analyses.

The expression profiling and survival analysis of ST6GalNAc-I, MUC5AC, VCAN, and NECTIN2 were performed using TCGA-LUAD. For the TCGA survival analysis, TCGA-LUAD gene expression values were calculated based on the top 25% as high-expression and the bottom 25% as low-expression groups. Further, GSEA was performed using high versus low expression of MUC5AC and ST6GalNAc-I. Protein-protein interaction network analysis was carried out using the STRING database.

### Statistics.

The data were analyzed for statistical significance with 2-tailed *t* test using GraphPad Prism 10.0.2 software. *P* less than 0.05 was considered statistically significant. All experiments were performed in triplicates at minimum. LMMs were used to look at changes in cell growth over time. Data were modeled on the natural log scale. The LMMs included fixed effects for group, time, and the group-time interaction, and time is modeled as a categorical variable. An autoregressive order 1 covariance structure was assumed for the analysis. Westfall stepdown method was used to adjust pairwise comparisons for multiple comparisons (84). SAS software version 9.4 (SAS Institute Inc.) was used for analysis of cell growth data.

### Study approval.

The mouse studies were performed in accordance with the US Public Health Service *Guide for the Care and Use of Laboratory Animals* (National Academies Press, 2011) under a protocol approved by the Institutional Animal Care and Use Committee, UNMC.

### Data availability.

RNA sequence data for this study were deposited at the NCBI’s Gene Expression Omnibus under accession numbers GSE290957 and GSE290456. A Supporting Data Values file is available online as supplemental material.

## Author contributions

IL, SKB, and AKG conceived and designed the experiments. MIA, SC, AS, and IL performed the experiments. MIA, SC, AS, GN, ZWA, SKB, AKG, and IL curated data. IL, SKB, and AKG acquired funding. MIA, SC, AS, GN, ZWA, PK, DDS, MWN, and IL collected data. MIA, SC, AS, MWN, SKB, AKG, and IL devised methodology. IL, SKB, and AKG provided resources. ZWA performed all bioinformatics analyses. SML and LMS provided pathological analysis and statistics. The manuscript was written by IL and MIA with input from SKB and AKG and reviewed by all authors. MIA, SC, AS, GN, ZWA, PK, DDS, SML, LMS, MWN, SKB, AKG, and IL reviewed and edited the manuscript. All authors read and approved the final manuscript.

## Supplementary Material

Supplemental data

Unedited blot and gel images

Supporting data values

## Version 1 05/15/2025

Electronic publication
