# Supplementary material for: ST6GalNAc-I regulates tumor cell sialylation via NECTIN2/MUC5AC-mediated immunosuppression and angiogenesis in non–small cell lung cancer
Source: J Clin Invest. 2025 May 15;135(10):e186863. doi: 10.1172/JCI186863 (PMC12077904; doi:10.1172/JCI186863)
Supplement: Supplemental data [file jci-135-186863-s046.pdf]

Supplemental Figures and Tables

ST6GalNAc-I regulates tumor cell sialylation via NECTIN2/MUC5AC-mediated immunosuppression and angiogenesis in non-small cell lung cancer

Muthamil Iniyan Appadurai[1], Sanjib Chaudhary[1], Ashu Shah[1], Gopalakrishnan Natarajan[1], Zahraa Wajih Alsafwani[1], Parvez Khan[1], Dhananjay D Shinde[2], Subodh M Lele[3], Lynette Smith[4], Mohd Wasim Nasser[1,5], Surinder K. Batra[1, 5*], Apar Kishor Ganti[1,5,6,7*], Imayavaramban Lakshmanan[1,5*]

***Corresponding Author**

[1]Department of Biochemistry and Molecular Biology, University of Nebraska Medical Center, Omaha, NE 68198-5870, USA

[2]Department of Pathology, Microbiology, and Immunology, University of Nebraska Medical Center, Omaha, Nebraska, USA

[3]Department of Pathology and Microbiology, University of Nebraska Medical Center, Omaha, NE, 68198-1850, USA.

[4]Department of Biostatistics, College of Public Health, University of Nebraska Medical Center, Omaha, NE, 68198-4375, USA.

[5]Fred & Pamela Buffett Cancer Center University of Nebraska Medical Center, Omaha, NE 68198-5870, USA.

[6]Department of Internal Medicine, University of Nebraska Medical Center, Omaha, NE, 68198-1850, USA.

[7]Division of Oncology-Hematology, Department of Internal Medicine, VA Nebraska Western Iowa Health Care System, NE, 68105-1850, USA.

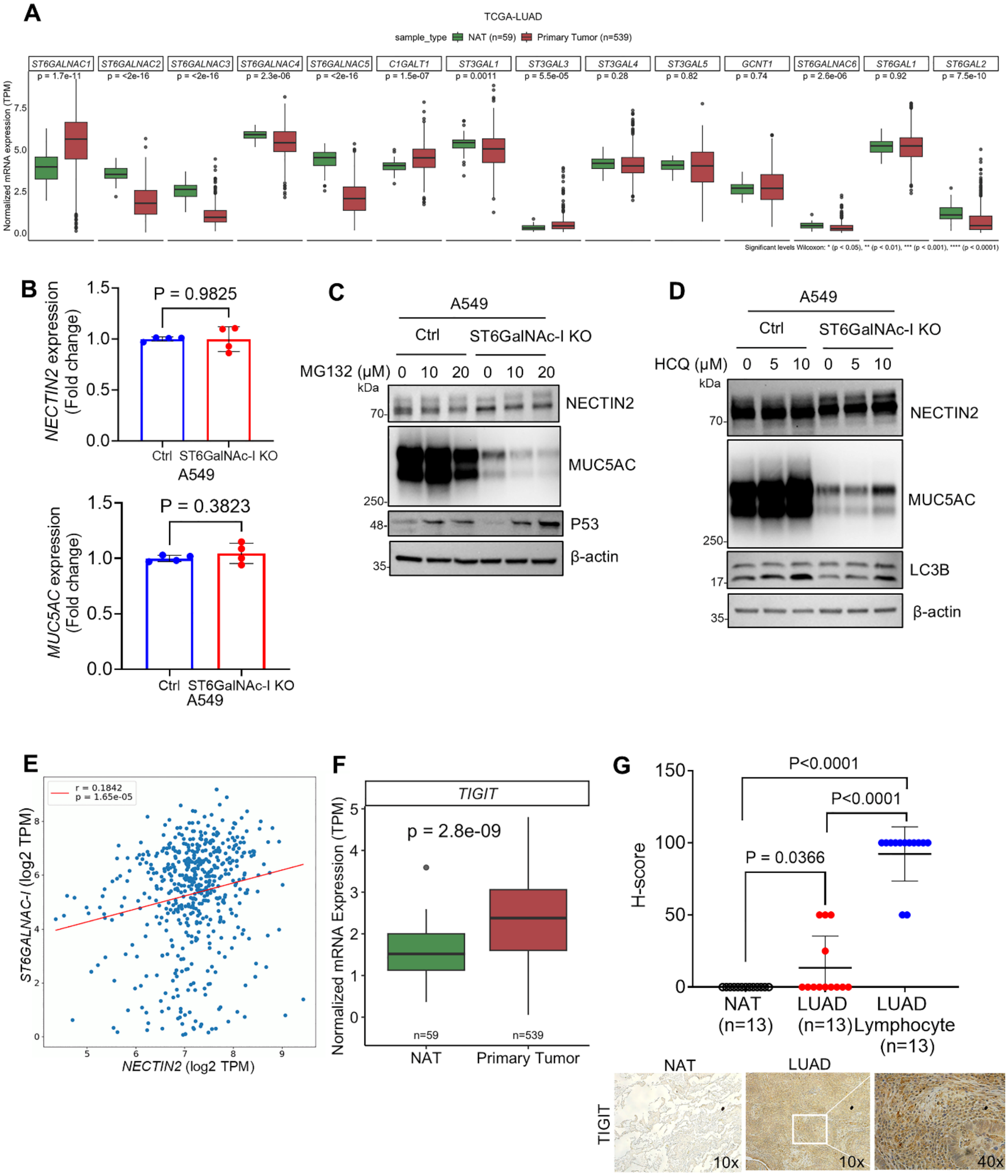
A
TCGA-LUAD
sample_type NAT (n=59) Primary Tumor (n=539)
ST6GALNAC1 p = 1.7e-11
ST6GALNAC2 p = <2e-16
ST6GALNAC3 p = <2e-16
ST6GALNAC4 p = 2.3e-06
ST6GALNAC5 p = <2e-16
C1GALT1 p = 1.5e-07
ST3GAL1 p = 0.0011
ST3GAL3 p = 5.5e-05
ST3GAL4 p = 0.28
ST3GAL5 p = 0.82
GCNT1 p = 0.74
ST6GALNAC6 p = 2.6e-06
ST6GAL1 p = 0.92
ST6GAL2 p = 7.5e-10
Normalized mRNA expression (TPM)
Significant levels Wilcoxon: * (p < 0.05), ** (p < 0.01), *** (p < 0.001), **** (p < 0.0001)
B
P = 0.9825
NECTIN2 expression (Fold change)
Ctrl ST6GalNAc-I KO
A549
P = 0.3823
MUC5AC expression (Fold change)
Ctrl ST6GalNAc-I KO
A549
C
A549
Ctrl ST6GalNAc-I KO
MG132 (μM) 0 10 20 0 10 20
kDa
NECTIN2
MUC5AC
P53
β-actin
D
A549
Ctrl ST6GalNAc-I KO
HCQ (μM) 0 5 10 0 5 10
kDa
NECTIN2
MUC5AC
LC3B
β-actin
E
r = 0.1842
p = 1.65e-05
ST6GALNAC-I (log2 TPM)
NECTIN2 (log2 TPM)
F
TIGIT
p = 2.8e-09
Normalized mRNA Expression (TPM)
n=59 n=539
NAT Primary Tumor
G
P<0.0001
P<0.0001
P = 0.0366
H-score
NAT (n=13)
LUAD (n=13)
LUAD Lymphocyte (n=13)
NAT LUAD
TIGIT
10x 10x 40x

Supplemental Figure 1. Comparison of sialyltransferase expression and normal lung tissues and ST6GalNAc-I/NECTIN2/TIGIT expression in LUAD. (**A**) Bar diagram represents that expression of ST6GalNAc-I and other glycosyltransferaseas in LUAD and normal tissue adjacent to the tumor (NAT). (**B**) Quantitative RT-PCR analysis shows *NECTIN2* and *MUC5AC* expression in A549 ST6GalNAc-I KO and control cells. Significance was determined by a two-tailed t-test (n=4). (**C**) Immunoblot showing the expression of NECTIN2, MUC5AC, and p53 following proteasome inhibitor, MG132, treatment (0, 10 and 20 μM) for 48 h in A549 ST6GalNAc-I KO and control lysates. (**D**) Immunoblot showing the expression of NECTIN2, MUC5AC, and LC3B following lysosomal inhibitor, HCQ, treatment (0, 5, and 10 μM) for 48 h in A549 ST6GalNAc-I KO and control lysates. β-actin was used as a control. (**E**) Correlation of *ST6GalNAc-I* and *NECTIN2* in LUAD. (**F**) Overexpression of *TIGIT* in LUAD using LUAD-TCGA. (**G**) Immunohistochemical analysis indicated that TIGIT is overexpressed in LUAD lymphocytes. Significance was determined by a two-tailed t-test (n=13). Magnification: 10x and 40x.

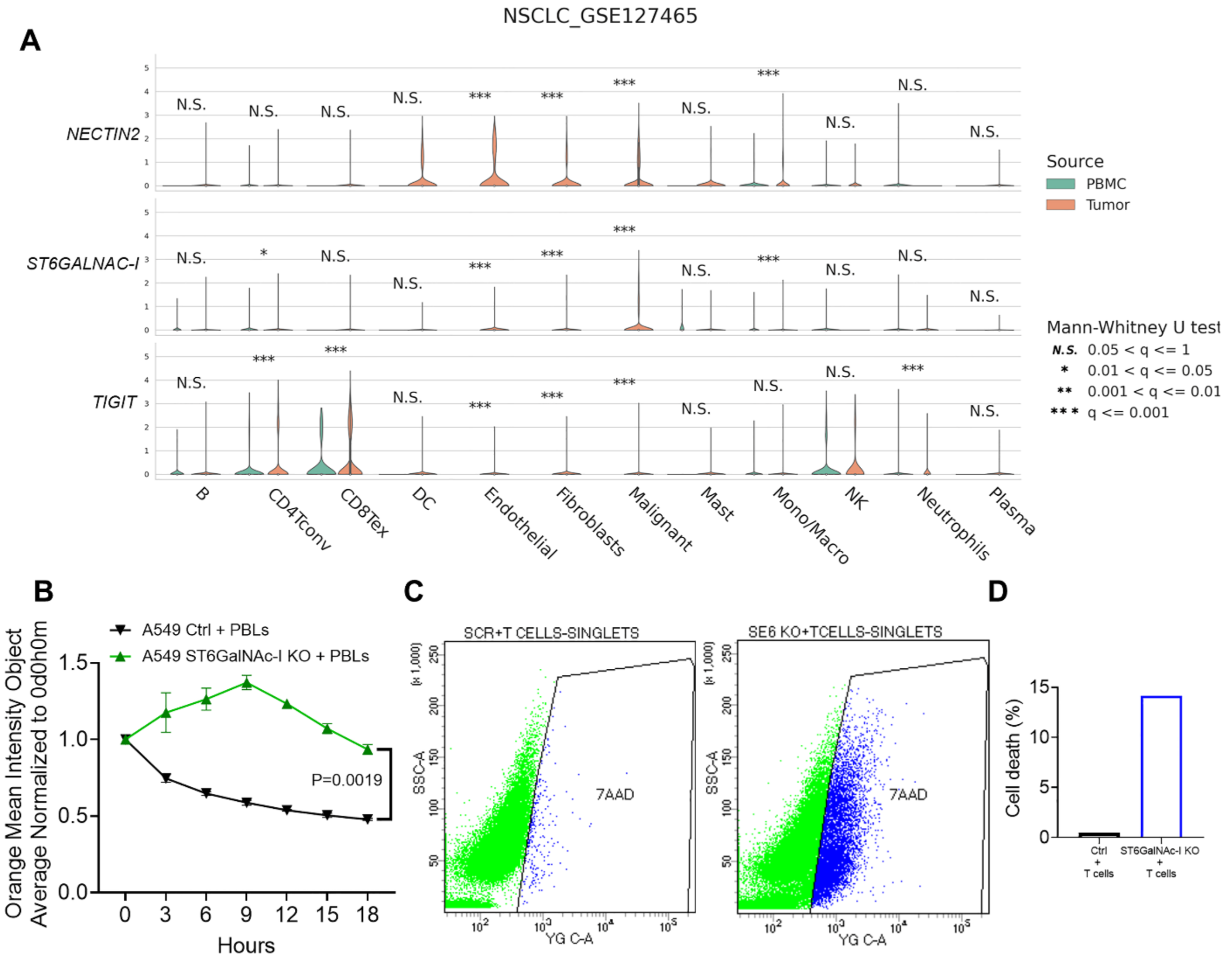

Supplemental Figure 2. In silico single-cell data analysis and T cell-mediated tumor cell killing assay in A549 cells and metabolomic pathways of P-DMEA. (**A**) Single-cell data analysis indicated that TIGIT is expressed in T cells. (**B**) Killing of A549 ST6GalNAc-I KO cells were significantly high when co-culture with PBLs compared to control cells with PBLs as demonstrated by IncuCyte live imaging with CytoTox Red assay. Data were analyzed by Student’s t-test. (**C** and **D**) FACS-based LIVE/DEAD staining data showed increased apoptotic activity in ST6GalNAc-I KO cells when co-culture with T cells from the pooled cell populations.

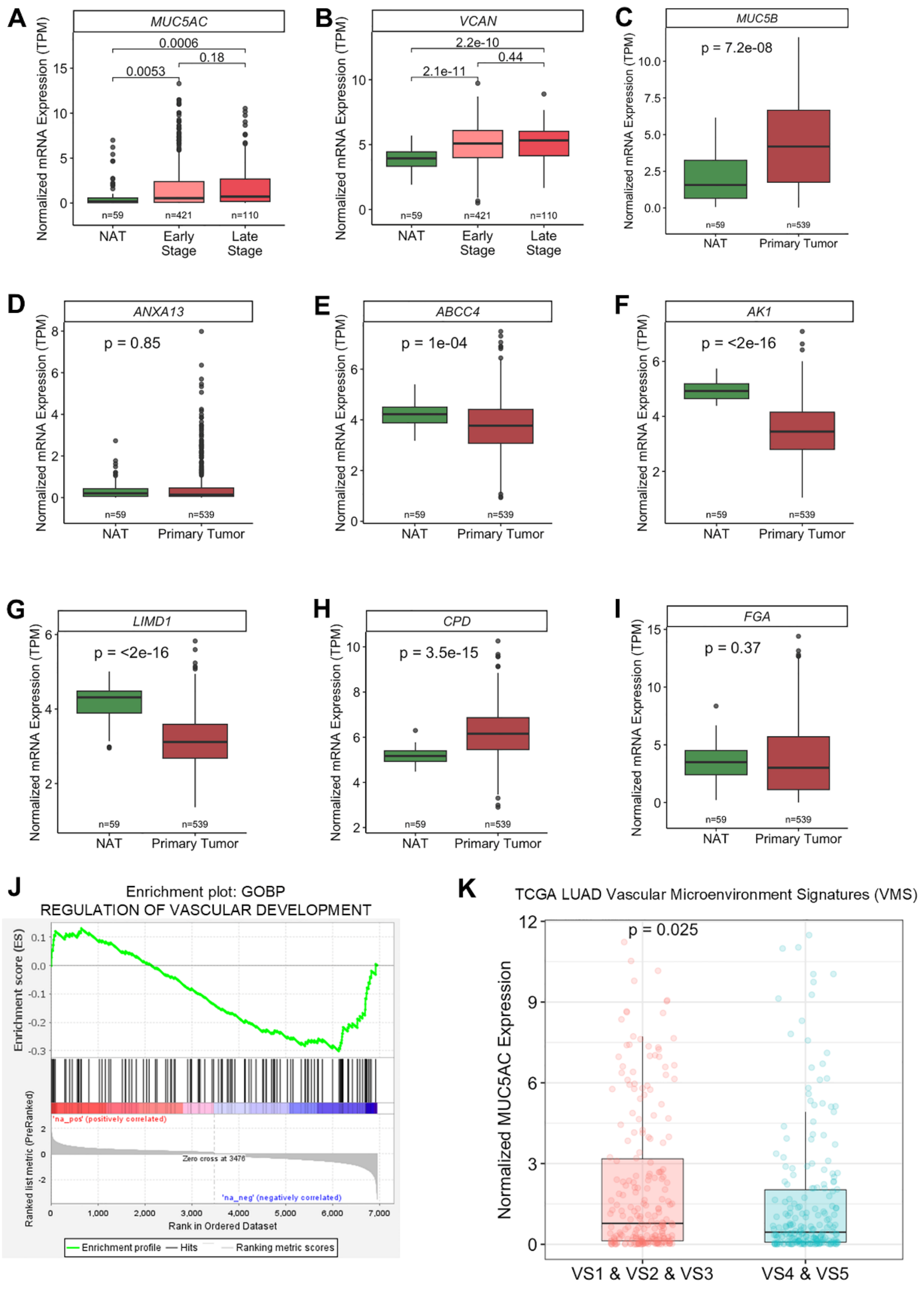
A
MUC5AC
0.0006
0.18
0.0053
Normalized mRNA Expression (TPM)
n=59
n=421
n=110
NAT
Early Stage
Late Stage
B
VCAN
2.2e-10
0.44
2.1e-11
C
MUC5B
p = 7.2e-08
n=539
Primary Tumor
D
ANXA13
p = 0.85
E
ABCC4
p = 1e-04
F
AK1
p = <2e-16
G
LIMD1
p = <2e-16
H
CPD
p = 3.5e-15
I
FGA
p = 0.37
J
Enrichment plot: GOBP
REGULATION OF VASCULAR DEVELOPMENT
Enrichment score (ES)
Ranked list metric (PreRanked)
'na_pos' (positively correlated)
Zero cross at 3476
'na_neg' (negatively correlated)
Rank in Ordered Dataset
Enrichment profile
Hits
Ranking metric scores
K
TCGA LUAD Vascular Microenvironment Signatures (VMS)
p = 0.025
Normalized MUC5AC Expression
VS1 & VS2 & VS3
VS4 & VS5

Supplemental Figure 3. Validation of ST6GalNAc-I/MUC5AC associated genes in LUAD. (**A** and **B**) Analysis of *MUC5AC* and *VCAN* mRNA expression in the TCGA dataset. (**C-I**) The mRNA expression of *MUC5B, ANXA13, ABCC4, AK1, LIMD1, CPD*, and *FGA* in LUAD was evaluated using TCGA-LUAD dataset. (**J**) Gene set enrichment analysis (GSEA) shows that *MUC5AC* KD genes were significantly associated with vasculature development. (**K**) In silico analysis shows MUC5AC-high expressing patients associated with the LUAD vascular microenvironment gene signatures (VEGF receptor high, E-cadherin low) as compared to low (VEGFA receptor low, E-cadherin high) expression group. VS1: down CDH1 (E-cadherin) and up endothelial cell surface receptors, growth factors, SNAI1, and SNAI2. VS2: up VEGFA. VS3: up NOTCH3. VS4 up CDH1. VS5: down VEGFA and up endothelial cell surface receptors and various growth factors.

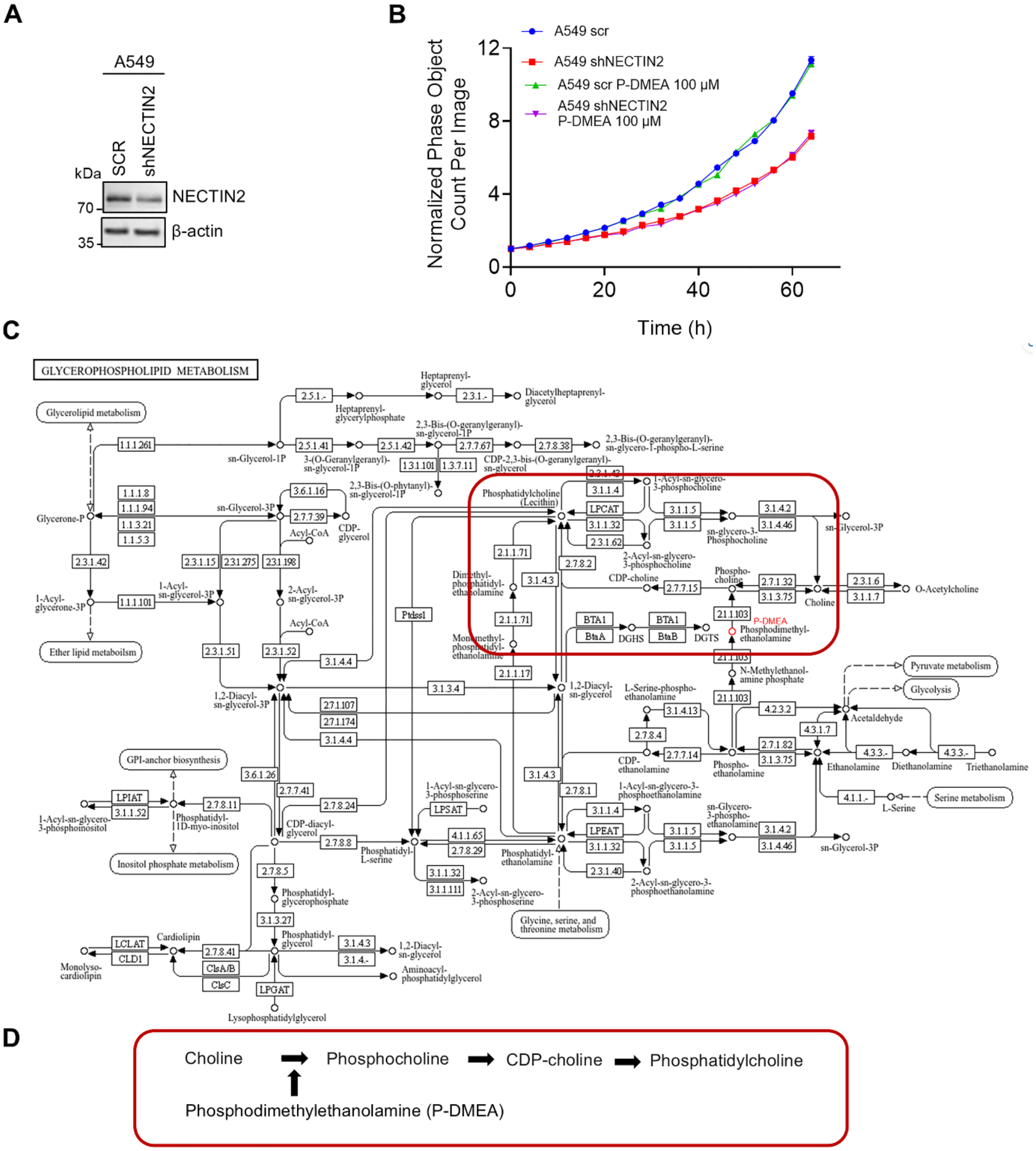
A
A549
SCR
shNECTIN2
kDa
70
35
NECTIN2
β-actin
B
A549 scr
A549 shNECTIN2
A549 scr P-DMEA 100 μM
A549 shNECTIN2 P-DMEA 100 μM
Normalized Phase Object Count Per Image
0
4
8
12
0
20
40
60
Time (h)
C
GLYCEROPHOSPHOLIPID METABOLISM
Glycerolipid metabolism
Ether lipid metaboilsm
GPI-anchor biosynthesis
Inositol phosphate metabolism
Glycine, serine, and threonine metabolism
Pyruvate metabolism
Glycolysis
Serine metabolism
Phosphatidylcholine (Lecithin)
Choline
Phospho-choline
CDP-choline
O-Acetylcholine
P-DMEA
Phosphodimethyl-ethanolamine
N-Methylethanol-amine phosphate
Phosphatidyl-ethanolamine
Ethanolamine
Diethanolamine
Triethanolamine
Acetaldehyde
L-Serine
Cardiolipin
Lysophosphatidylglycerol
D
Choline
Phosphocholine
CDP-choline
Phosphatidylcholine
Phosphodimethylethanolamine (P-DMEA)

Supplemental Figure 4. P-DMEA-associated pathways. (**A**) Immunoblot shows knockdown of NECTIN2 in A549 cells. (**B**) IncuCyte-based proliferation following P-DMEA treatment (100 μM) in A549 Scr and shNECTIN2 cells. (**C**) KEGG pathway analysis shows glycerophospholipid metabolism, indicating that the P-DMEA-associated pathway is involved in phosphatidylcholine biosynthesis indicated in the red box. (**D**) P-DMEA pathway in phosphatidylcholine biosynthesis.

A

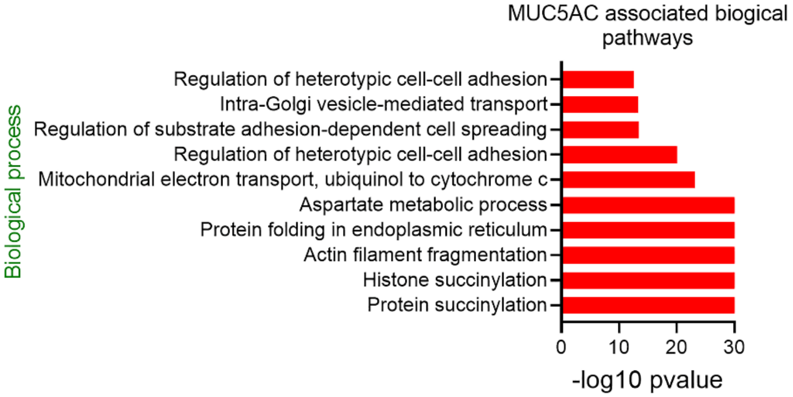

B

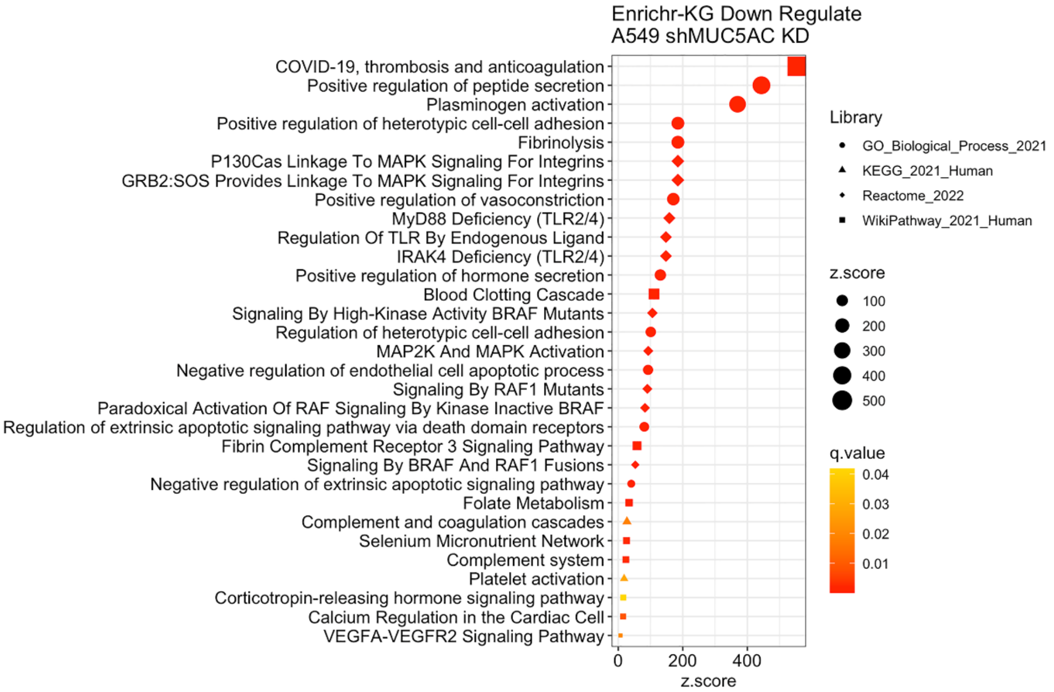

Supplemental Figure 5. MUC5AC associated pathways in LUAD. (**A** and **B**) Gene ontology-based biological pathway analysis using MUC5AC KD associated molecules.

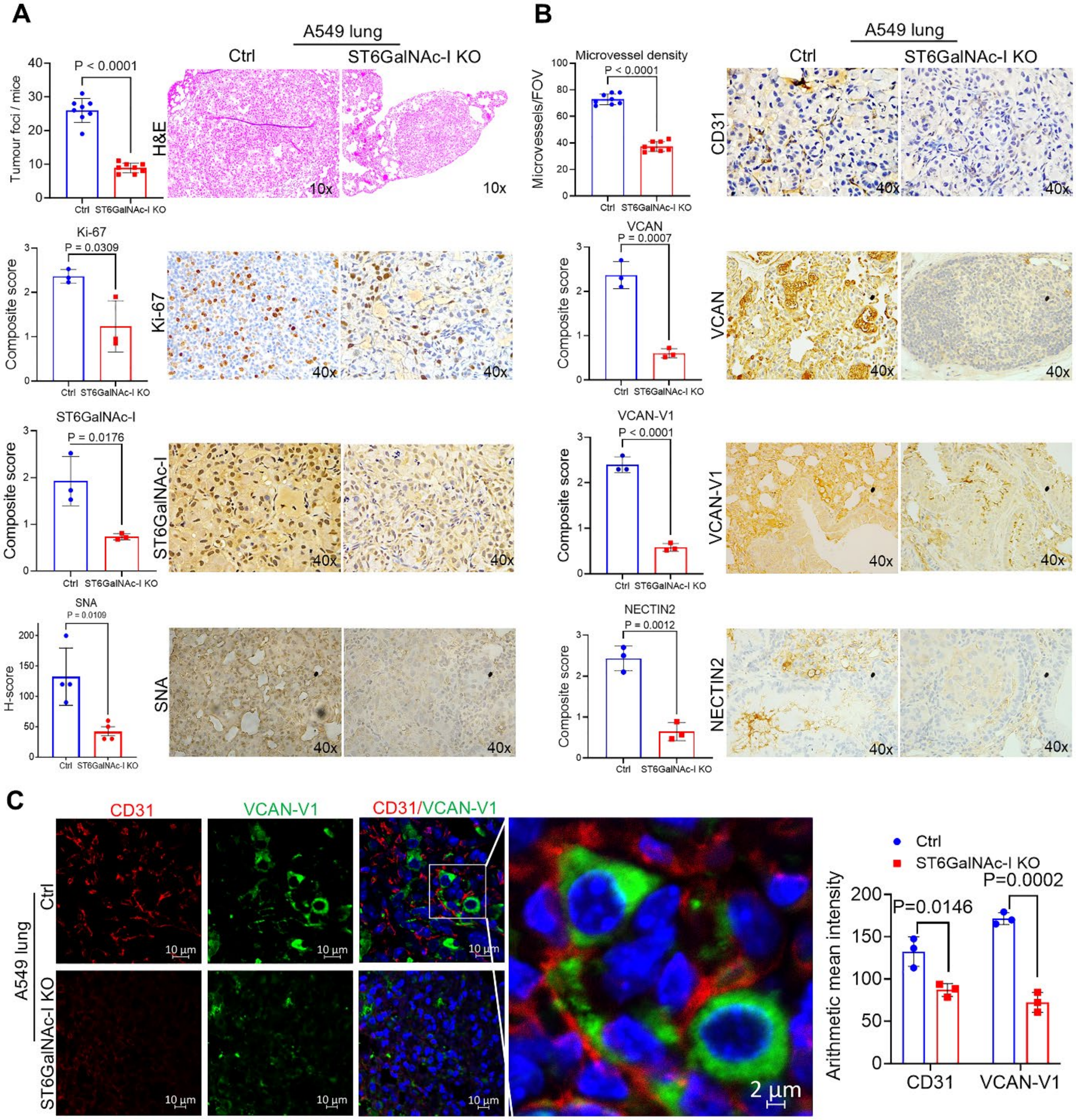

A
B
C
A549 lung
Ctrl
ST6GalNAc-I KO
Tumour foci / mice
P < 0.0001
H&E
10x
Ki-67
P = 0.0309
Composite score
40x
ST6GalNAc-I
P = 0.0176
SNA
P = 0.0109
H-score
Microvessel density
P < 0.0001
Microvessels/FOV
CD31
VCAN
P = 0.0007
VCAN-V1
P < 0.0001
NECTIN2
P = 0.0012
CD31/VCAN-V1
10 μm
2 μm
Arithmetic mean intensity
P=0.0146
P=0.0002

Supplemental Figure 6. ST6GalNAc-I is associated with tumor development and angiogenesis. (**A** and **B**) A549 ST6GalNAc-I KO cells injected mice had significantly reduced lung tumor foci as compared to respective control cells injected mice (n=8). Expression of Ki-67, ST6GalNAc-I, SNA, CD31, VCAN, VCAN-V1, and NECTIN2 were significantly reduced in ST6GalNAc-I KO lung tumors compared to control tumors. (**B**, upper panel) Quantification of the microvessel density was done using CD31 stained tumor sections in eight fields for each tumor section and presented as a mean number per field (0.2 mm^2). Significance was determined by a two-tailed t-test (n=3). Magnification: 10x for H&E and 40x for IHC. (**C**) Co-localization of VCAN-V1 and CD31 in ST6GalNAc-I KO xenograft lung tissues. Significance was determined by a two-tailed t-test (n=3). Scale bars: 10 μm and 2 μm.

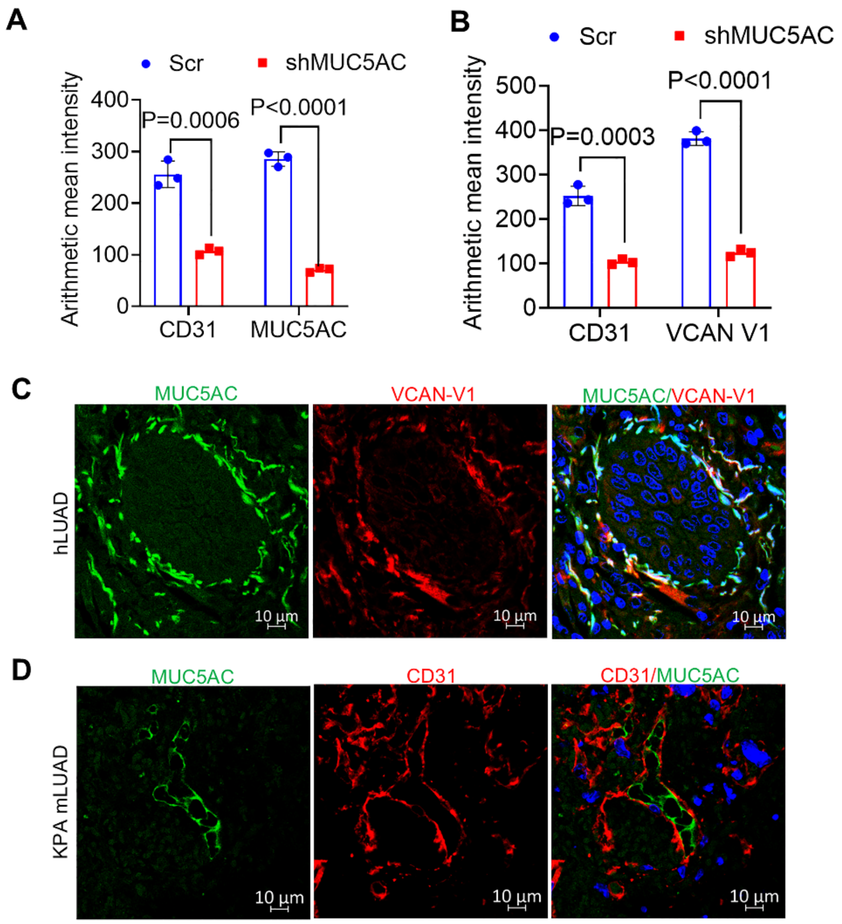

Supplemental Figure 7. Co-expression of MUC5AC/VCAN-V1 in LUAD. (**A** and **B**) Quantification of CD31 with MUC5AC and VCAN-V1 expression in MUC5AC knockdown xenograft shown in Figure 8 D&E. Data were analyzed by Student's t-test (n=3). (**C** and **D**) Co-localization of MUC5AC and VCAN-V1 in human NSCLC tumor and CD31 with MUC5AC in genetically engineered KPA mouse tumor. Scale bars: 10 µm.

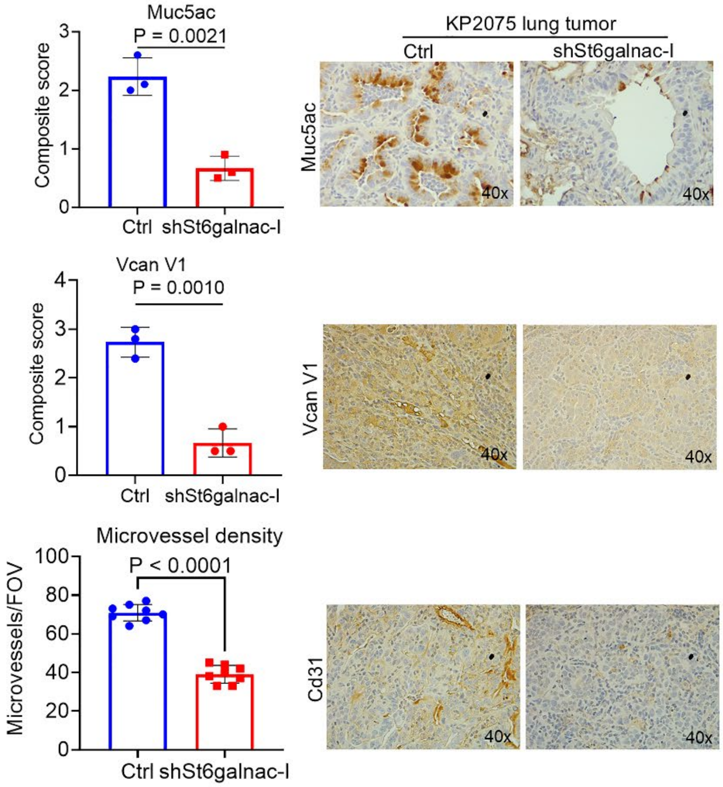

Supplemental Figure 8. Immunohistochemical analysis for MUC5AC, VCAN-V1, and CD31 in St6galnac-I knockdown lung tumor (C57BL/6 mice). KP2075 St6galnac-I knockdown cells injected mice showed reduced staining of Muc5ac, Vcan V1, and microvessel density. Quantification of the microvessel density was done using Cd31 stained tumor sections in eight fields for each tumor section and presented as a mean number per field (0.2 mm^2). Significance was determined by a two-tailed t-test (n=3). Magnification: 40x.

Supplemental Figure 9. ST6GalNAc-I and MUC5AC-induced LUAD liver metastasis. (**A** and **B**) A549 ST6GalNAc-I KO or MUC5AC KD cells injected mice showed reduced liver metastasis incidence compared to respective control cells injected mice. Data were analyzed by Student's t test (n=4). Magnification: 40x. (**C** and **D**) Trichrome staining indicated that A549 ST6GalNAc-I KO or MUC5AC KD-derived lung tumors showed reduced tumor matrix. Data were analyzed by Student's t-test (n=3). Magnification: 10x.

Supplemental Table 1. Metabolomic analysis. List of metabolites significantly changed in A549 ST6GalNAc-I KO (n=5) compared to A549 control cells (n=5).

Metabolite name	Formula	Calc. MW	Mass-to-charge ratio	Retention time (min)	CSID	Reference Ion
noradrenaline	C8 H11 N O3	169.0741	170.0814	2.218	926	[M+H]+1
3-Methoxytyramine	C9 H13 N O2	167.0953	168.1025	2.353	1606	[M+H]+1
1-(4-Hydroxy-3,5-dimethoxyphenyl)-1,2,3-propanetriol	C11 H16 O6	244.0939	243.0866	4.655	35013334	[M-H]-1
5-hydroxyxanthotoxin	C12 H8 O5	232.0362	233.0436	10.647	4532375	[M+H]+1
DL-Glutamine	C5 H10 N2 O3	146.0691	147.0764	9.224	718	[M+H]+1
P-DMEA	C4 H12 N O4 P	169.0509	170.0582	11.557	133469	[M+H]+1
Eglumetad	C8 H11 N O4	185.0694	186.0767	10.581	184747	[M+H]+1
gamma-Glu-gln	C10 H17 N3 O6	275.112	276.1193	10.589	133013	[M+H]+1
beta-D-Galp-(1->3)-D-GalpNAc	C14 H25 N O11	383.1441	422.1073	8.931	390021	[M+K]+1
Nicotinamide	C6 H6 N2 O	122.048	123.0552	1.797	911	[M+H]+1
Creatinine	C4 H7 N3 O	113.0592	114.0665	3.069	568	[M+H]+1
3-Methylsulfolene	C5 H8 O2 S	132.0248	133.0321	6.664	64103	[M+H]+1
3-Tropanol	C8 H15 N O	141.1156	124.1123	2.934	13871816	[M+H-H2O]+1
2-Pyrrolidone	C4 H7 N O	85.05296	86.06023	8.958	11530	[M+H]+1
Hypoxanthin	C5 H4 N4 O	136.039	137.0463	4.059	768	[M+H]+1
Glycerol 3-phosphate	C3 H9 O6 P	172.0144	173.0216	9.932	734	[M+H]+1
N-benzoyl-4-hydroxyanthranilic acid	C14 H11 N O4	257.0683	258.0758	4.956	139764	[M+H]+1
DL-Carnitine	C7 H15 N O3	161.1058	162.1129	8.58	282	[M+H]+1
L-(-)-Threonine	C4 H9 N O3	119.0586	120.0659	8.752	6051	[M+H]+1
Niacin	C6 H5 N O2	123.0321	124.0393	3.594	913	[M+H]+1
DL-Arginine	C6 H14 N4 O2	174.1111	175.1184	12.893	227	[M+H]+1

Supplemental Table 2. Cell line panel. Mutational background of non-small cell lung cancer cell lines along with endothelial and normal bronchial epithelial cells.

Cells	Tumor type	Mutation status			
		K-Ras	p53	EGFR	Others
**Human cell lines**					
HBEC	Normal Lung	WT	WT	WT	-
HULEC5a	Lung microvascular endothelial	WT	WT	WT	-
HUVEC	Umbilical vein endothelial	WT	WT	WT	-
A549	LUAD	G12S	WT	WT	KEAP1 (G333C)
NCI-H1437	LUAD	WT	R267P	WT	TGFBR1 (R487W); MAP2K1 (Q56P)
NCI-H292	LUAD	G12S	WT	WT	-
NCI-H1975	LUAD	WT	R273H	L858R/T790M	PIK3CA (G118D)
NCI-H2122	LUAD	G12C	C176F/Q16L	WT	-
SW1573	LUAD	G12C	WT	WT	PIK3CA (K111E); CTNNB1 (S33F)
NCI-H3122	LUAD	WT	E285V	WT	EML4-ALK fusion
HCC827	LUAD	WT	V218del	E746_A750del	-
HCC4006	LUAD	WT	Y205H	L747_E749del	-
PC9	LUAD	WT	R248Q	E746_A750del	MUC5AC (C3226Ter)
NCI-H23	LUAD	G12C	M246I	WT	-
NCI-H2030	LUAD	G12C	G262V	WT	-
H1650	LUAD	WT	c.673-2A>G	E746_750del	-
**Mouse cell lines**					
KP2075	LUAD	G12D	R172H	WT	-

NOTE: Mutation status of each cell line in KRAS, p53, EGFR and others was determined from the Sanger Catalogue of Somatic Mutations in Cancer database (COSMIC, http://www.sanger.ac.uk/genetics/CGP/cosmic/); DepMap (https://depmap.org/portal/) and Cellosaurus (https://www.cellosaurus.org/index.html). Abbreviation: WT, wild-type.
