# Supplementary material for: ST6GalNAc-I regulates tumor cell sialylation via NECTIN2/MUC5AC-mediated immunosuppression and angiogenesis in non–small cell lung cancer
Source: J Clin Invest. 2025 May 15;135(10):e186863. doi: 10.1172/JCI186863 (PMC12077904; doi:10.1172/JCI186863)

Full unedited blot for Figure 2 E

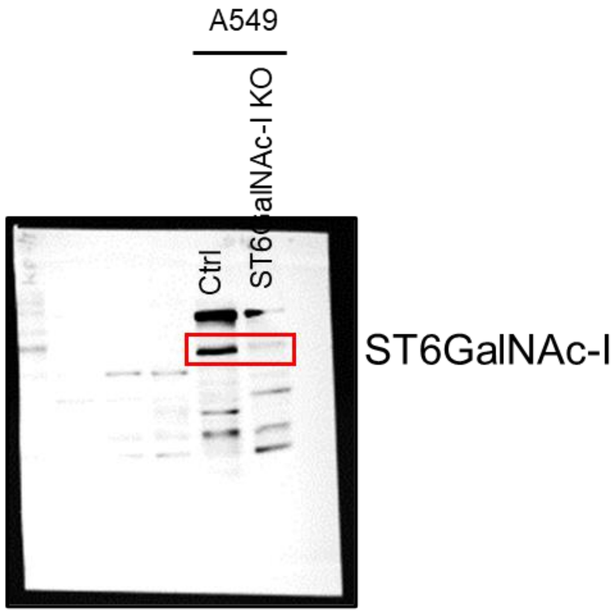

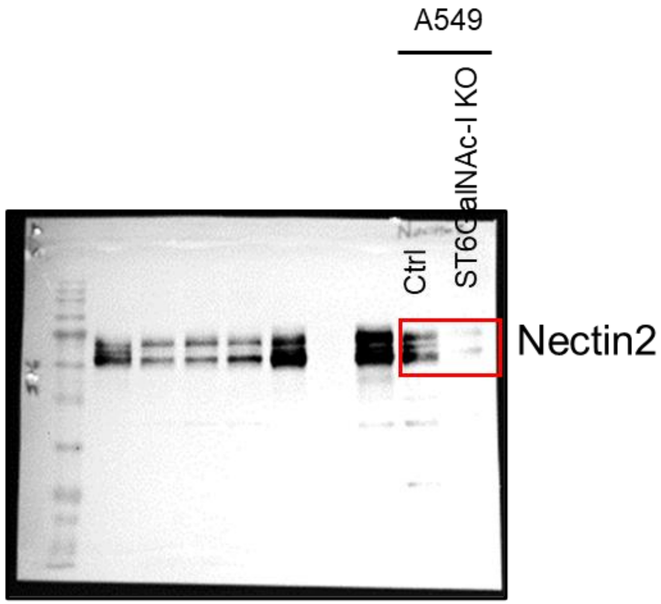

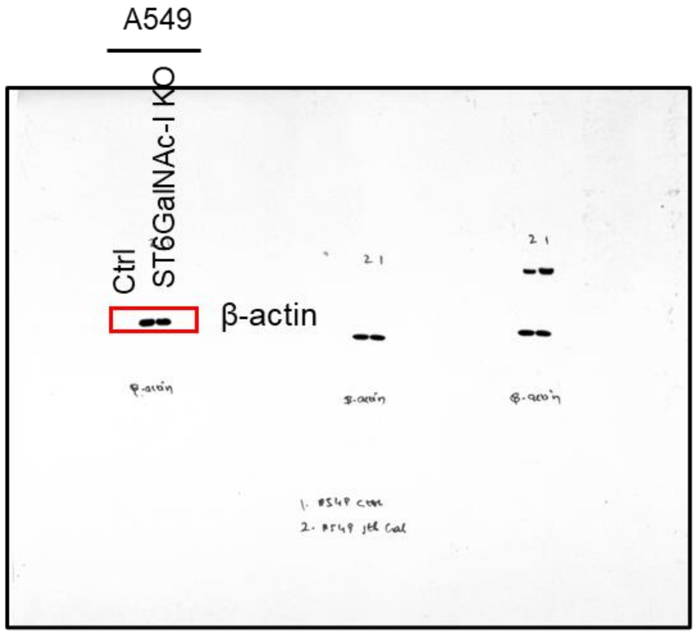

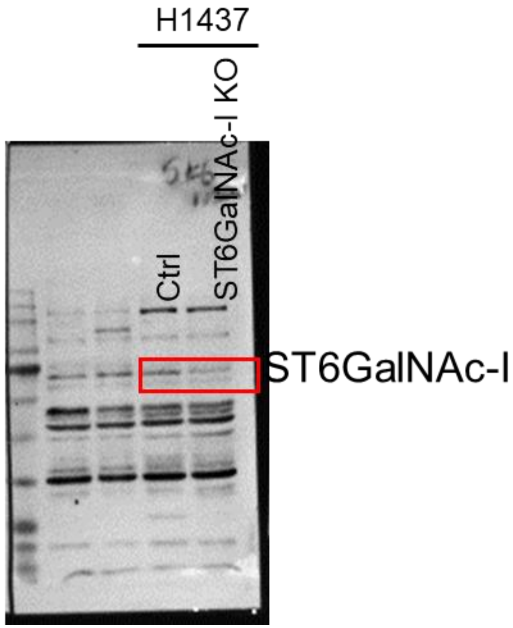

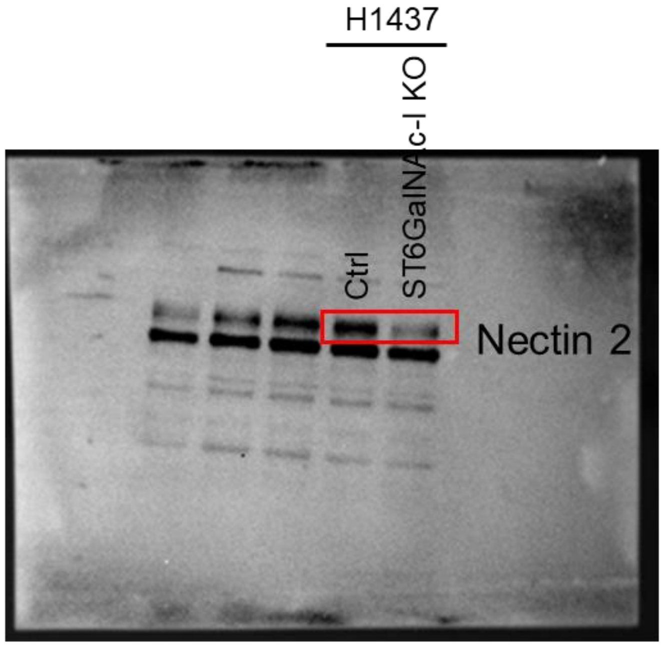

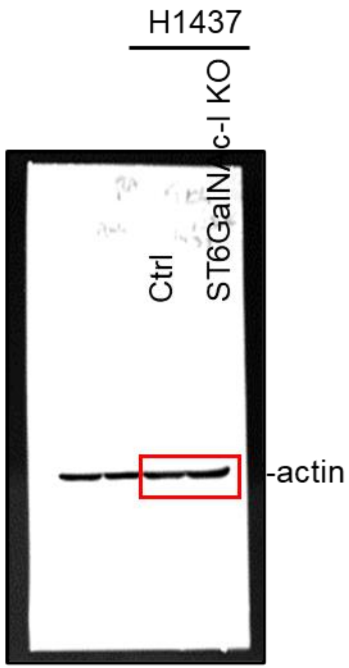

Full unedited blot

Figure 2 F

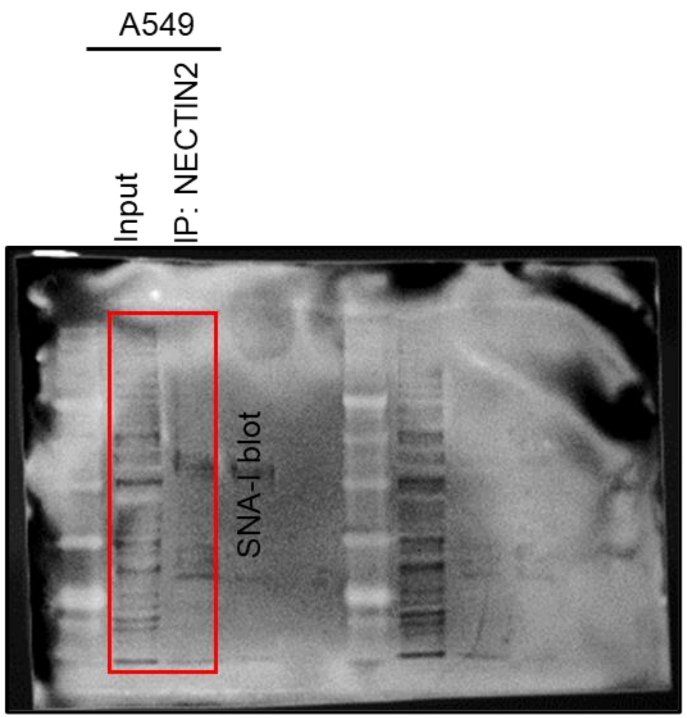

Figure 2 G

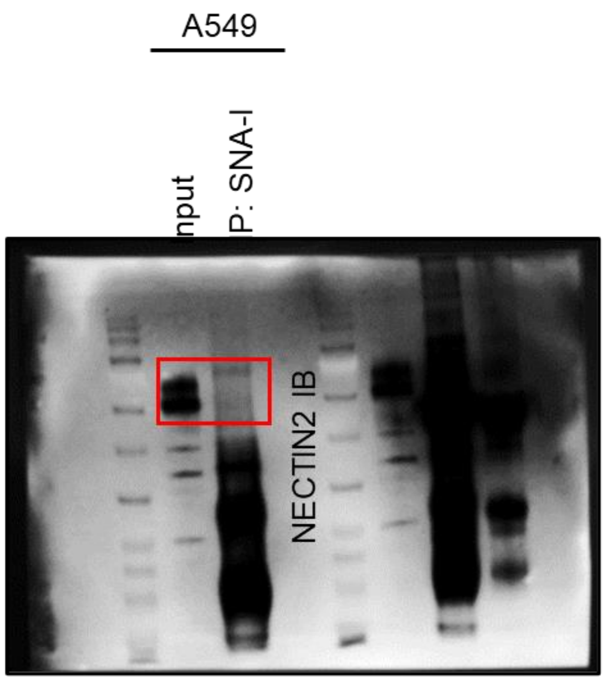

Figure 2 H

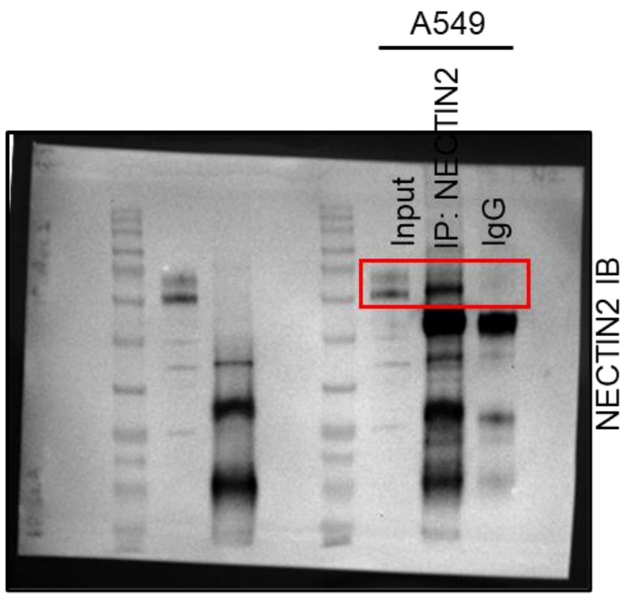

Full unedited blot

Figure 3 H

A549 T-cell

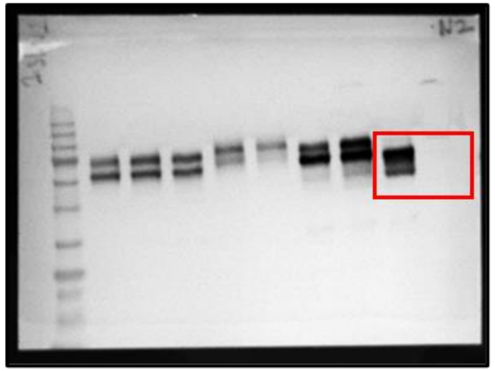

NECTIN2 IB

Figure 3 H

A549 T-cell

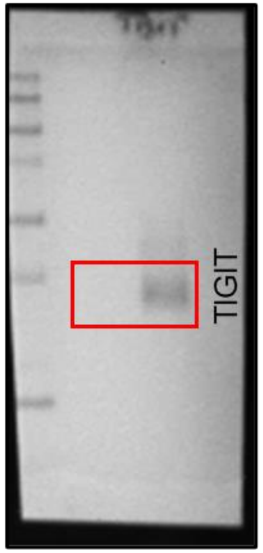

TIGIT

Figure 3 H

A549 T-cell

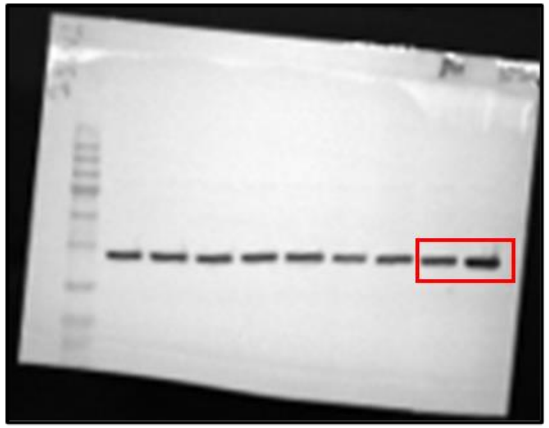

β-actin

Full unedited blot

Figure 3 I

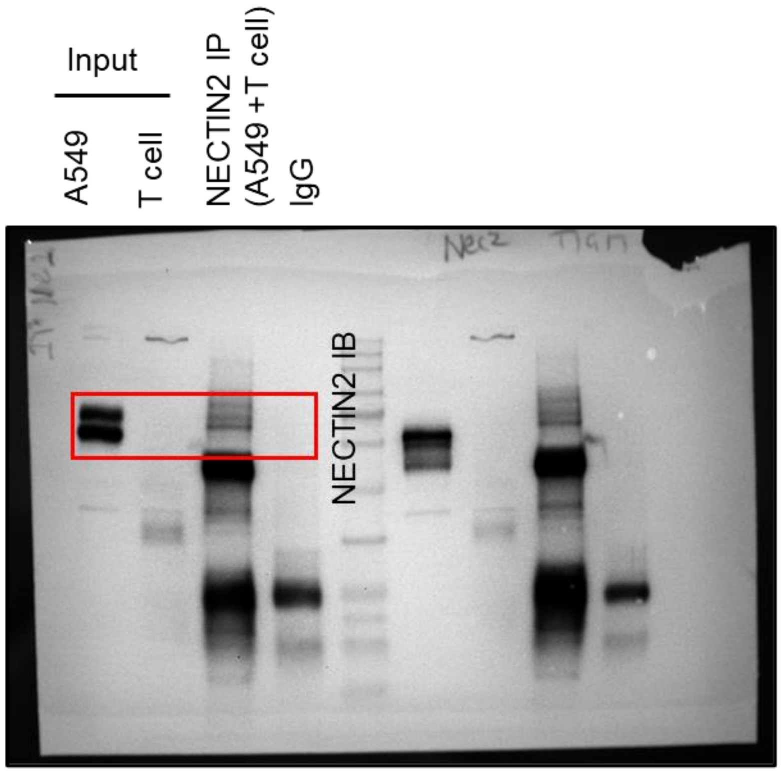

Figure 3 I

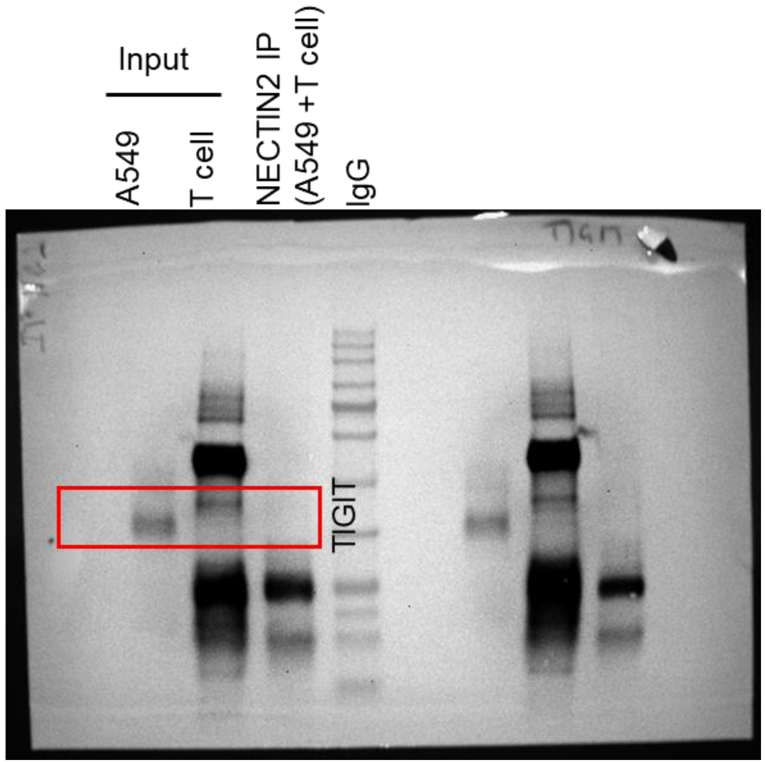

Full unedited blot

Figure 4 B

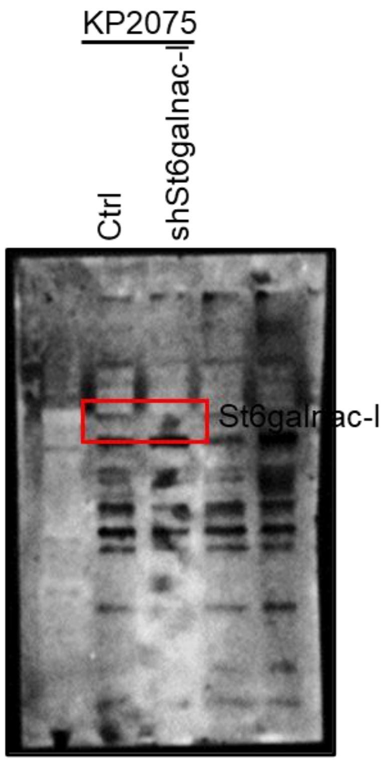

Figure 4 B

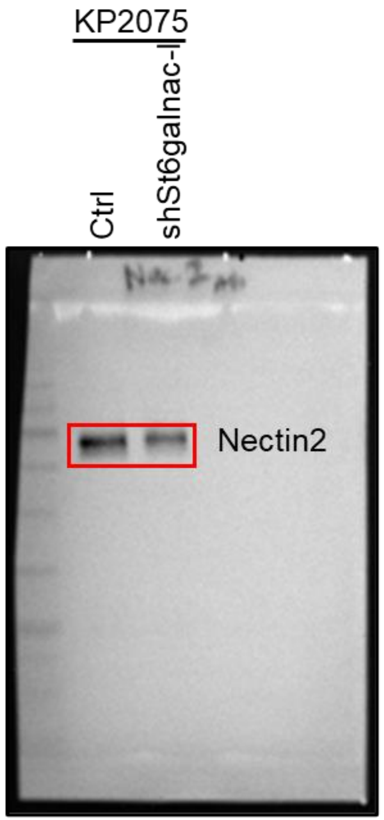

Figure 4 B

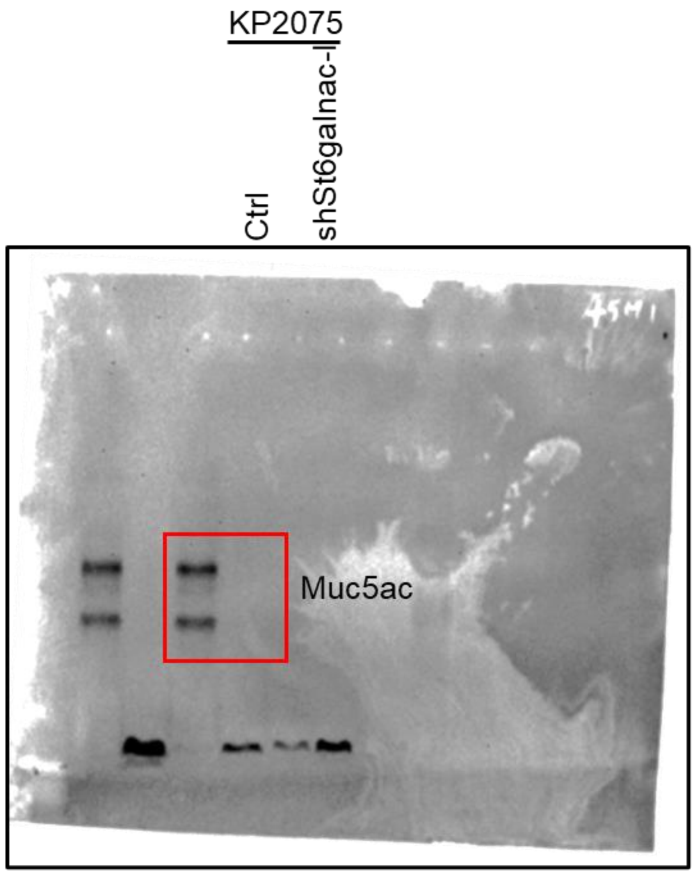

Figure 4 B

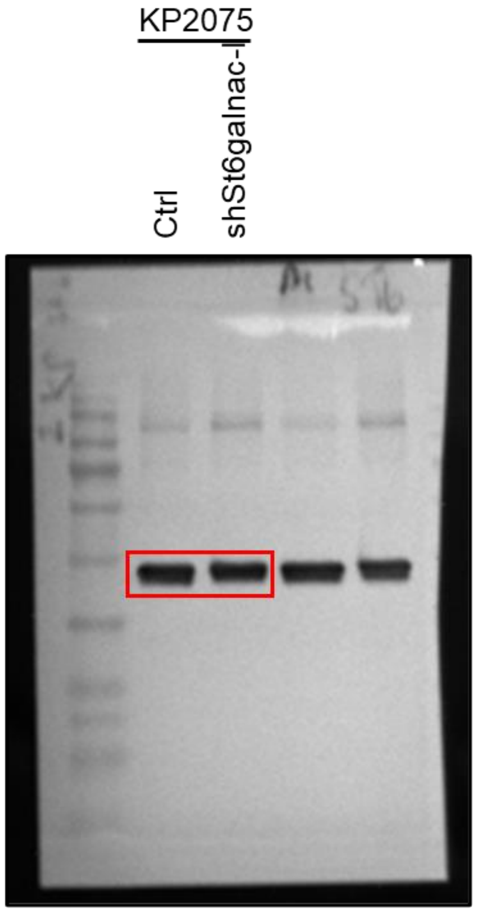

Full unedited blot Figure 5 E

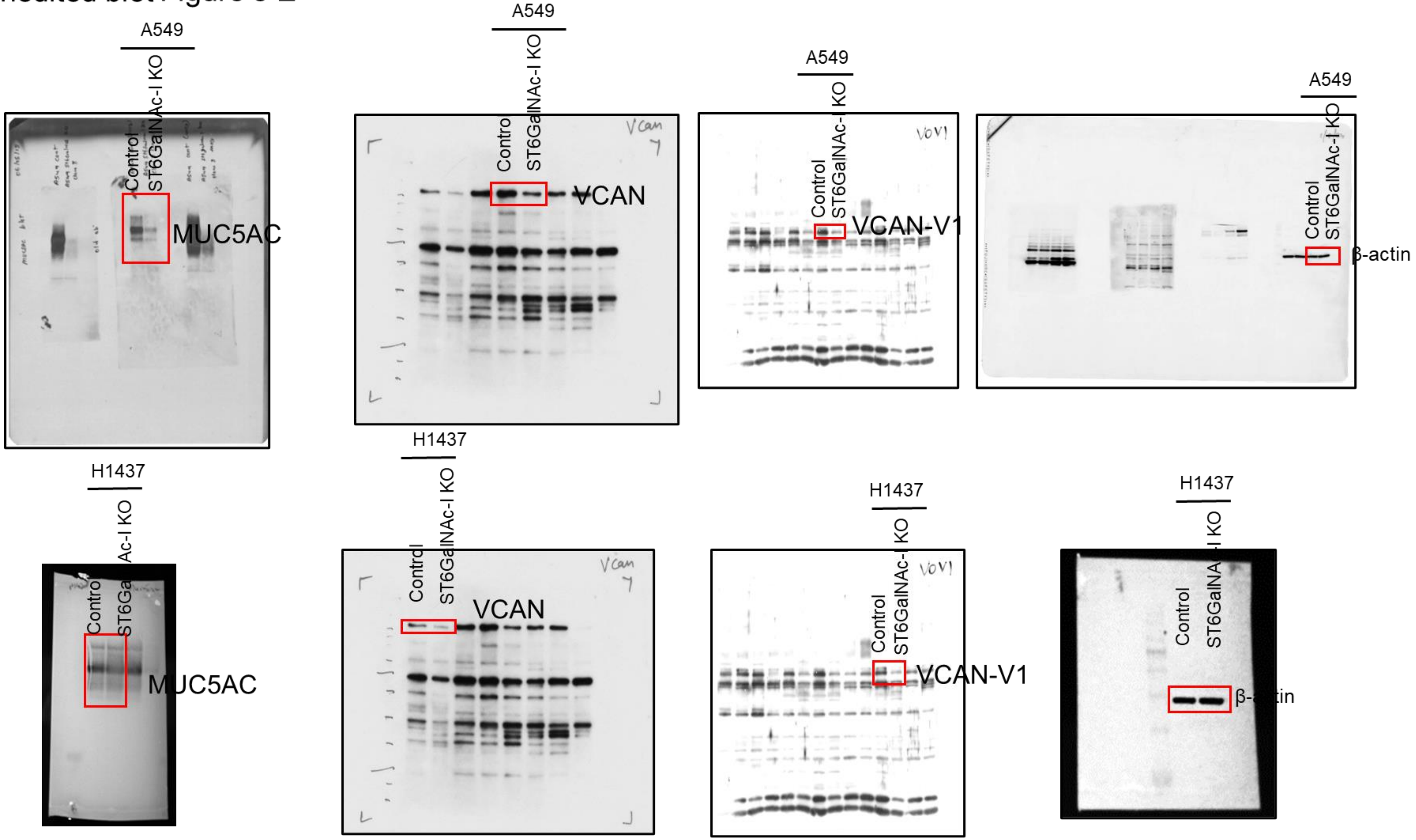

Full unedited blot Figure 5 F

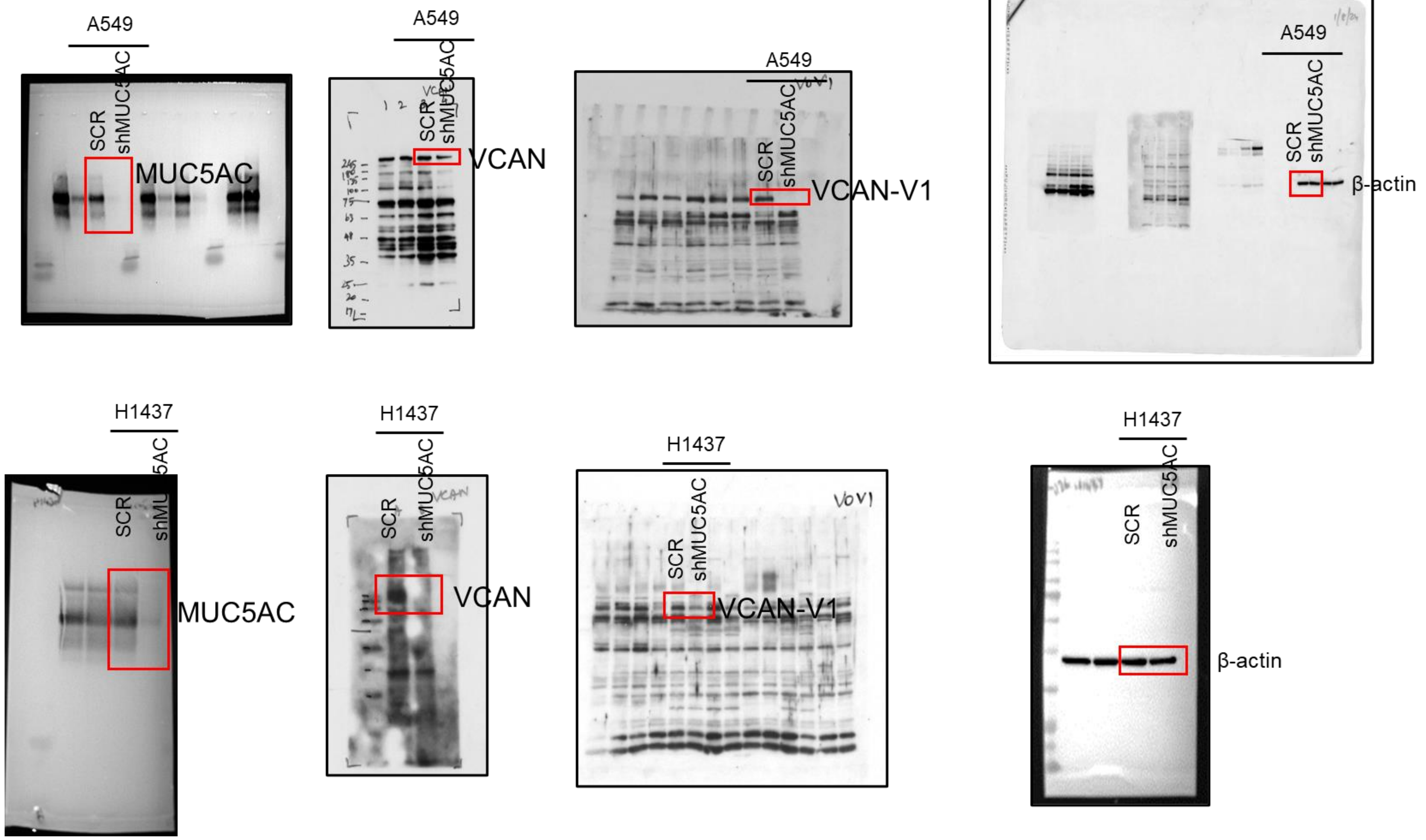

Full unedited blot

Figure 5 H

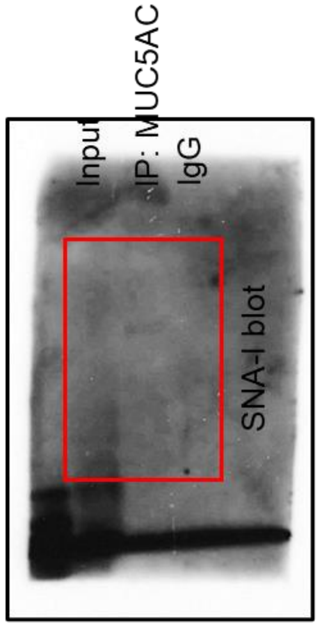

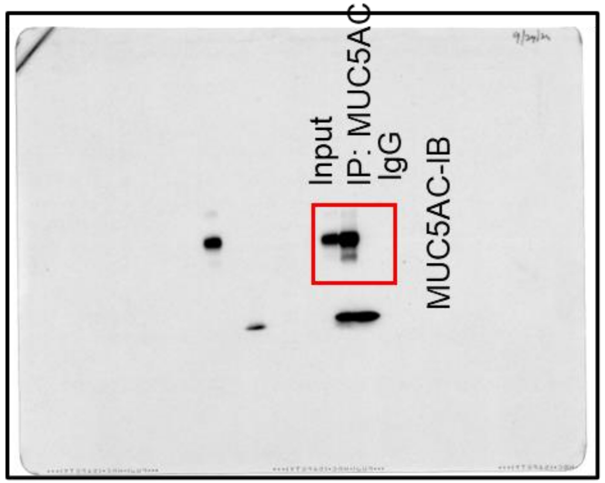

Figure 5 I

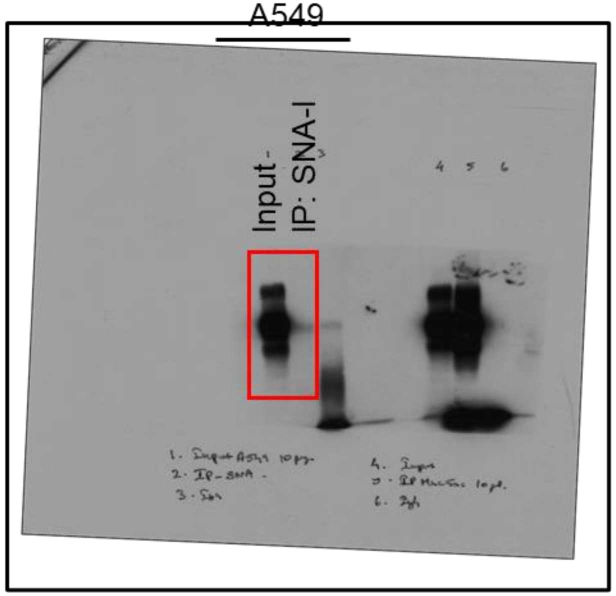

Full unedited blot Figure 6 B

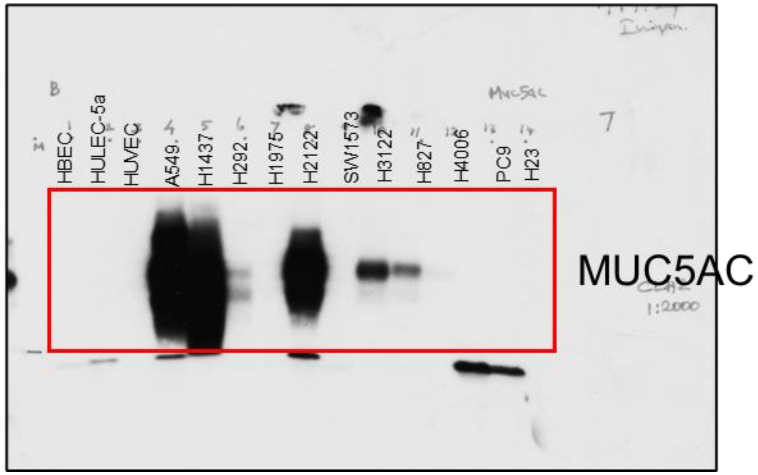

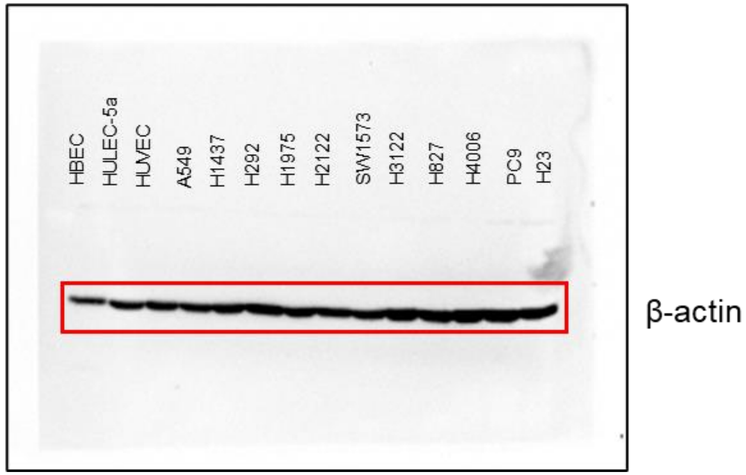

Full unedited blot

Figure 7 D

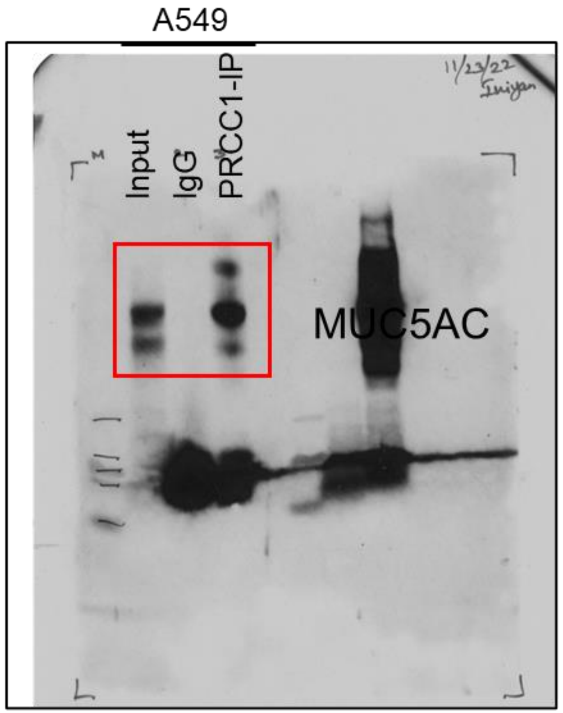

Figure 7 E

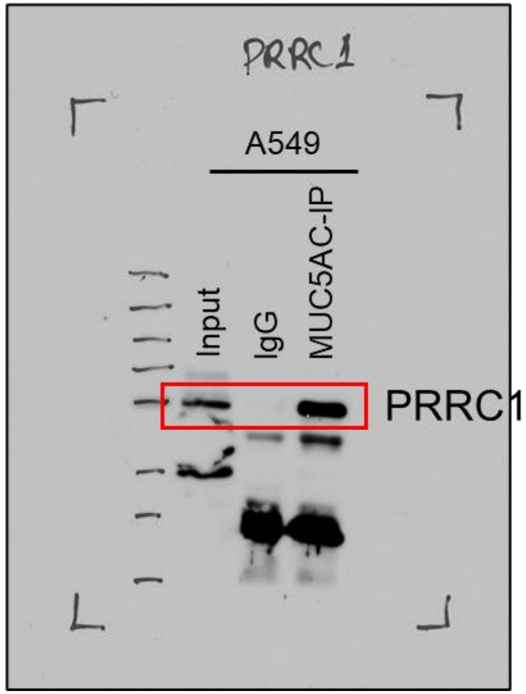

Full unedited blot

Supplementary Figure 1 C

A549

Ctrl ST6GalNAc-I KO

MG132 (μM) 0 10 20 0 10 20

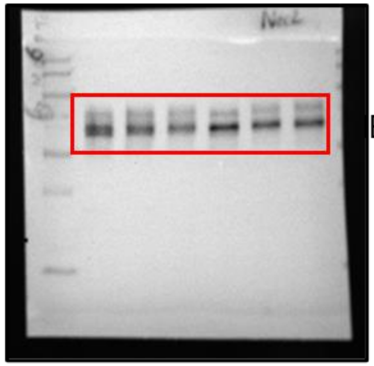

ECTIN2

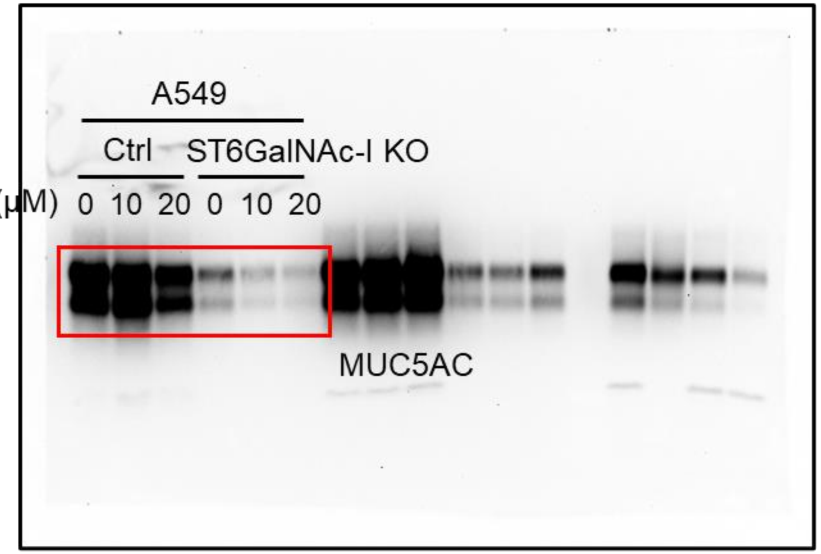

A549

Ctrl ST6GalNAc-I KO

MG132 (μM) 0 10 20 0 10 20

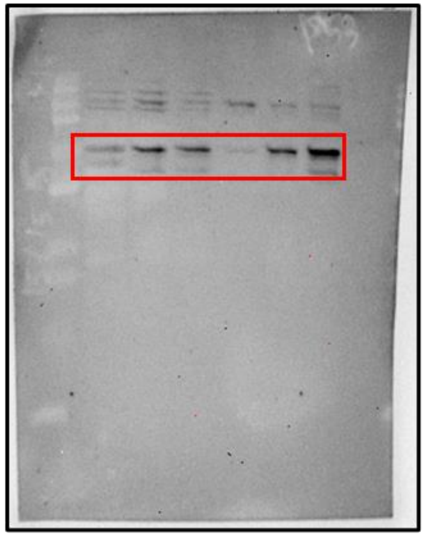

A549

Ctrl ST6GalNAc-I KO

MG132 (μM) 0 10 20 0 10 20

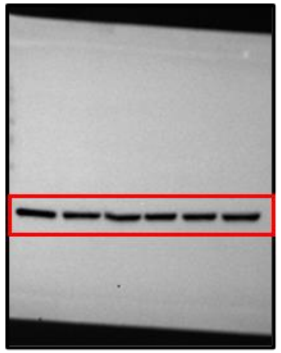

β-actin

Full unedited blot

Supplementary Figure 1 D

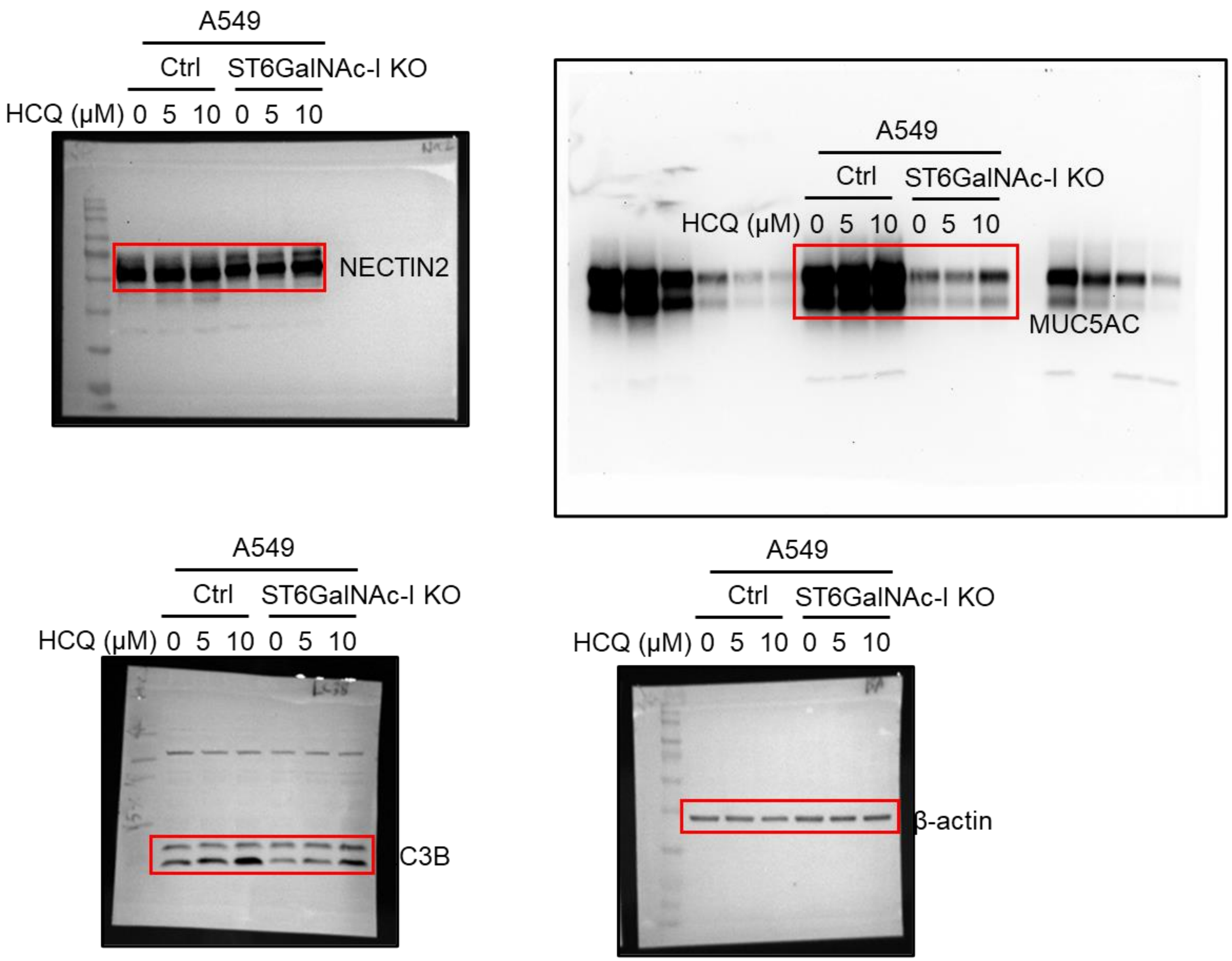

Full unedited blot

Supplementary Figure 4 A

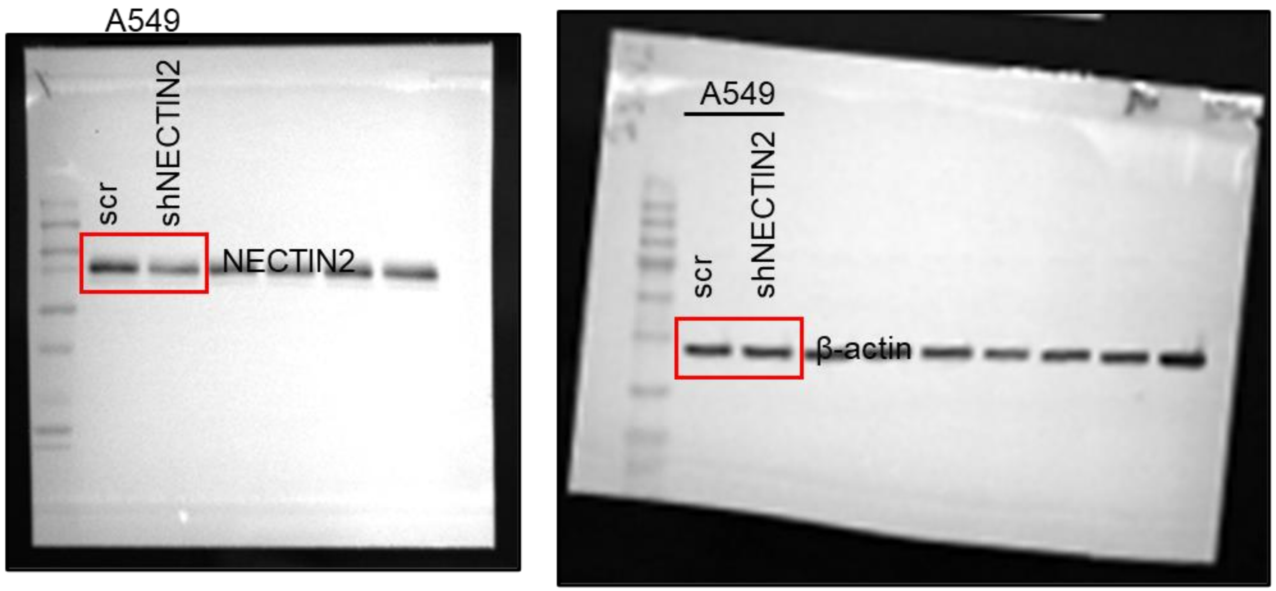

Supplement: Unedited blot and gel images [file jci-135-186863-s047.pdf]
